# Annual Academy of Sickle Cell and Thalassaemia (ASCAT) conference: a summary of the proceedings

**DOI:** 10.1186/s12919-020-00204-1

**Published:** 2020-12-16

**Authors:** Crawford Strunk, Andrew Campbell, Raffaella Colombatti, Biree Andemariam, Rachel Kesse-Adu, Marsha Treadwell, Baba P. D. Inusa

**Affiliations:** 1 ProMedica Russell J. Ebeid Children’s Hospital, 2142 North Cove Blvd, Toledo, OH 43606 USA; 2 Center for Cancer and Blood Disorders, Children’s National Medical Center, 111 Michigan Avenue, NW, Washington, DC 20010 USA; 3 Clinic of Pediatric Hematology-Oncology, Department of Woman’s and Child’s Health, University of Padua, 35129 Padua, Italy; 4 New England Sickle Cell Institute, University of Connecticut Health, 263 Farmington Ave, Farmington, CT 06030 USA; 5 Guy’s and St. Thomas’ NHS Foundation Trust, Westminster Bridge Road, London, SE1 7EH UK; 6 Department of Hematology/Oncology, UCSF Benioff Children’s Hospital Oakland, 747 52nd Street, Oakland, CA 94609 USA; 7 Department of Haematology, Evelina London Children’s Hospital and Guy’s and St. Thomas’ NHS Foundation Trust, Westminster Bridge Road, London, SE1 7EH UK

**Keywords:** Sickle cell disease, Thalassaemia, ASCAT conference, Blood transfusion, Bone marrow transplantation, Anaemia

## Abstract

The fourteenth annual ASCAT conference was held 21–23 October 2019. The theme of the conference was ‘Sickle Cell and Thalassaemia disorders new treatment horizon; while ensuring patient safety and delivering excellence in routine patient care.’ Over the three-day conference, topics on current and novel models of care, advances in bone marrow transplant and gene therapy, as well as the psychosocial aspects of mind, body and health related quality of life were discussed. In addition, blood transfusion, apheresis, iron chelation therapy and acute haemolytic complications were presented. Quality standards in the diagnosis and treatment of sickle cell and thalassaemia were reviewed. Experts from Europe, the United Kingdom, the Middle East, the United States and Africa reported up-to-date scientific data, guides to comprehensive care, and current research into developing cures and advancing current therapy were described. In addition, oral and poster presentations on novel research from all over the world were shown during the conference.

## Highlights

- The conference provided updates in treatment of thalassaemia and sickle cell disease
- Advances in gene therapy, gene editing and bone marrow transplant strategies were presented
- Strategies to minimize non-adherence and enhance coping skills were detailed
- Epigenetic and genetic global electronic patient databases were discussed
- Chronic disease management and prognostic factors of disease severity were reviewed

## Background

The Annual Academy of Sickle Cell and Thalassaemia (ASCAT) Conference took place on October 21–23, 2019 at Etc Venues, County Hall, London, SE1 7 PB, UK. The theme of the conference this year was ‘Sickle cell and Thalassaemia disorders new treatment horizon; while ensuring patient safety and delivering excellence in routine patient care.’ The goal of the conference was to build on the previous annual meetings’ themes and address some of the areas that delegates suggested for further coverage such as the role of community staff in haemoglobinopathies and preconception counselling. In light of the emergence of new therapies on the horizon, comes complexities in decision-making, increasing cost, limitation in access and the need for new strategies to ensure a sustainability in provision [1, 2]. Sickle cell disease (SCD) and Thalassaemia are the leading disorders of haemoglobin referred to as haemoglobinopathies.

Ensuing disparities in access to new therapies for these disorders is not just limited to low-income settings as well as high-income countries, furthermore professional engagement may be lacking [3, 4]. This conference was targeted at physicians, specialist psychologists, nurses, scientists, and all relevant experts involved in the management of haemoglobinopathies. This year’s conference was given as one plenary session. Over three days, the conference sought to address several key topics, including:

- Implementation of newborn screening for haemoglobinopathies, cohort studies, and the development of management protocols and national control programmes.
- Novel therapies including update on Phase I/II/III clinical trials - in SCD and thalassaemia
- Update on curative therapies, including gene therapy, gene editing and bone marrow transplantation
- Psychosocial aspects including body, mind and health related quality of life in sickle cell disease and thalassaemia
- Blood transfusion including apheresis, iron chelation therapy and acute haemolytic complications.
- Global priorities in quality standards in the diagnosis and treatment of SCD and thalassaemia.
- ‘Meet the expert’ sessions, seminars and tutorials.
- A full patient forum involving children, adults and parents experience will be shared during the three days.

After welcoming delegates to the conference, Dr. Baba Inusa announced two planned joint Grand Rounds, the first with Evelina London on Monday 21st October and the second on the 23rd October session with Guy’s and St. Thomas NHS Foundation Trust to be presented by Drs Alexis Thompson, Lewis Hsu and Wale Atoyebi titled “Multidimensional approach of sickle cell disease.” In addition, a new initiative for 2019 was a combined EuroBloodNet and ASCAT joint patient forum sponsored by Novartis plc, the output of which is being presented at two international haematology meetings and prepared for peer publication.

### Day 1

#### Session 1 – Overview of Haemoglobinopathies

Speakers: Dr. David Rees, King’s College Hospital, London.

Dr. Paul Telfer, Barts Health NHS Trust, London.

Dr. Sebastian Lucas, Guy’s and St. Thomas’s NHS Foundation Trust, King’s College, London.

The introductory session of the conference discussed the pathophysiology of sickle cell disease (SCD) and thalassaemia. Dr. David Rees explored the current evidence of the pathophysiology of SCD, noting that the root cause of sickle cell disease is a mutated beta globin gene undergoing polymerisation in deoxygenated state. Evidence points to a single origin of HbS gene, suggesting it started in the Sudan, and spread by migration to the rest of Africa, the Middle East, and the Indian subcontinent. He also reviewed the natural history, epidemiology, and migratory patterns of SCD. Annual birth rates are highest in Africa and South/East Asia, and on the rise in North America, and Eastern Mediterranean. The disparity in patient survival in the west compared to sub-Saharan Africa was explored. Moreover life-expectancy in high-income countries ranges from 40 to 60 years for homozygous sickle cell disease. The clinical phenotype is also variable, especially as it relates to co-inheritance with other genetic factors, such as alpha thalassaemia or foetal haemoglobin co-inheritance as well as infections, environmental factors e.g. climate and air quality, and socioeconomic factors.

In his overview of thalassaemia, Dr. Paul Telfer reported on the clinical aspects and dilemmas of thalassaemia care and described the real-world evidence for the current standard treatment of thalassaemia. Dr. Telfer described a retrospective review of 70 transfusion dependent thalassaemia (TDT) patients over 12 years of age from nine centres undergoing current therapy. He reported that overall survival was 81.1% at age 50, with the main causes of death being myocardial, liver, infection, and malignancy. He also reported that 34% of patients from the UK are currently treated on combination chelation therapy despite a lack of studies. Non-transfusion dependent thalassaemia (NTDT) patients are reported to be at risk for thrombosis, pulmonary hypertension, and extramedullary haematopoiesis, suggesting that these patients may do better with blood transfusions starting at a young age in order to improve their quality of life. Results from studies of transplantation in thalassaemia have shown an event free survival (EFS) as high as 91%, while 20% of children have shown late effects complications including gonads and kidneys, but some may be secondary to prior disease process. Unlike in SCD, haploidentical transplantation has not been shown to be successful and is discouraged. However, gene therapy studies as well as Luspatercept have shown to be clinically beneficial in patients, leading to a reduction in transfusion requirements and sustained increase in haemoglobin.

Dr. Sebastian Lucas described the pathology and pathogenesis of SCD using morbid anatomy, gross and microscopic autopsy specimens. Dr. Lucas discussed the pathogenesis of acute and chronic organ failure, including the lungs, kidneys, spleen, SCD patients to determine correctly the cause of death and the extent of chronic disease. He emphasised the need to undertake more autopsies in the future and this will provide needed scientific understanding of major complications in SCD, hence benefitting patient care. Indeed, such recommendations for SCD patients is included in the autopsy guidelines of the Royal College of Pathologists.

#### Session 2 – Long-term Outcomes in SCD and Thalassaemia

Moderator: Dr. Winfred Wang, St. Jude Children’s Research Center, Memphis, TN.

Speakers: Dr. Mona El-Ghamrawy, Cairo University.

Dr. Winfred Wang.

Dr. Jennifer Knight-Madden.

Dr. Julie Makani, Muhumbili University, Tanzania.

Dr. Antonis Kattamis, National & Kapodistrian University of Athens, Greece.

This session reviewed the current evidence regarding long term outcomes of patients with SCD and thalassaemia.

Dr. Mona El-Ghamrawy discussed the results of a retrospective review of the patients at her institution, where the majority of patients have Hgb SS phenotype, but over 40% have sickle beta thalassaemia. There is no newborn screening programme in Egypt, and there are suboptimal referrals from outlying areas of the country. The average age at diagnosis is 4 years, and the median age at presentation is 2 years due to the absence of newborn screening. While almost 50% of patients have an affected sibling, just over 54% of siblings of affected children are routinely screened. The most common presentation is pallor and bone pain, while avascular necrosis, gallstones, central nervous system (CNS) events and iron overload were also common features. Patients’ adherence with transcranial Doppler (TCD) screening and hydroxycarbamide was 74%. There was no difference in the phenotypic severity between patients with HgbSS and Hgb S/ beta thalassaemia.

Dr. Winfred Wang presented partial results of the ongoing Sickle Cell Clinical Research and Intervention Program (SCCRIP), a retrospective cohort borne out of the Baby-HUG study with prospective data collection over 9 specific domains collected every two years into adulthood. Three of those domains, brain, kidney and bone, are reported here. Dr. Allison King’s investigation of hydroxycarbamide on neurocognition showed that in the realms of executive functioning, attention, and working memory, adolescents taking hydroxycarbamide did better than those who did not, with improvements noted in full scale intelligence quotient (FSIQ) as well. In the domain of kidneys, for those patients on hydroxycarbamide without elevated albumin: creatinine ratio (ACR) at the onset of therapy, 19/21 remained negative for microalbuminuria. In the 49% of patients who had microalbuminuria (MA) at baseline and had a positive ACR ratio, 1/3 improved with hydroxycarbamide therapy. Starting ACR ratio monitoring early, early hydroxycarbamide use, and addition of ACE inhibitor (ACEI) or angiotensin receptor blocker (ARB) in those who do not improve or develop MA on hydroxycarbamide was recommended. In addition, APOL1 screening may help identify patients who may be at increased risk of developing renal complications, so hydroxycarbamide or other reno-protective therapy may be started at a young age. Regarding the bone domain, low bone density scores are associated with osteonecrosis and chronic pain independently. This project seeks to develop and expand on need for DEXA imaging with prospective data.

Dr. Jennifer Knight-Madden presented updated data from the Jamaican SCD cohort. Although Jamaica has been a leader in sickle cell research, universal newborn screening only started in 2015, showing an incidence of 1 in 500 births. Simple interventions, including penicillin prophylaxis and splenic palpation, improved survival with mortality rates falling despite low hydroxycarbamide use. There is still an excess mortality of sickle cell patients compared to controls, with mean survival of age 40 for men, and 42 for women. For adults, the most common cause of death was acute chest syndrome followed by chronic organ complications. In young children, the most common cause of mortality was splenic sequestration, acute chest syndrome, sepsis and gastroenteritis. Evaluation of the data for middle-aged patients would occur at the next analysis.

Dr. Julie Makani discussed ‘Sickle in Africa: Consortium for Health, Education and Research on SCD in Africa.’ Of the 14 million children born with SCD between 2010 and 2050, only 10% will survive childhood; however, with basic care, including newborn screening and infection control, this improves to 50% without hydroxycarbamide or transplant. It is key to integrate health advocacy, research and training. While decentralising care to other centres, it remains critical to maintain and standardise comprehensive health care, health policy and guidelines, including improved access to blood transfusions, treatment of infections, imaging, and hydroxycarbamide, even though hydroxycarbamide is not readily accessible. In Africa, there is an absence of a multi-site cohort, consistent standards of care, human resources are low and there are few dedicated programs for SCD. However, patients are still demanding that providers explore curative treatment options. Because of the state of sickle cell care and research in Africa, the Sickle Pan-Africa Research Consortium (SPARCO) was founded with sites in Ghana, Nigeria, Tanzania, and a data collection site in South Africa. The goals of the consortium are to establish a registry, develop standards of care, short, medium- and long-term training, and research. Research goals include newborn screening implementation and hydroxycarbamide use. Working with the UK, training courses involving local patient communities at all levels is starting. Bone Marrow transplant programmes are being developed in Ghana, Nigeria and Tanzania. In addition, national and global governmental agencies, industry and philanthropic partnerships are being established, combining health care and research to cure and care.

Dr. Antonis Kattamis discussed long-term survival in thalassaemia. In the early days of treatment, only 25% of patients lived to be older than 10, but median survival was 40 years of age by 2000. Using patients from Thailand as an example, patients with the worst prognosis had survival of 51% at age 55; survival was better if they were treated at a major centre. Patients treated with standard therapy did better than those treated with BMT, although that difference was most likely the result of BMT toxicity. As adults, overall survival was the same between groups. In addition, it was noted that transplant type and degree of siderosis played a role in survival and toxicity. Causes of death have changed as patients survived from cardiac causes to liver failure, malignancy. The French registry of 702 patients showed a 20% mortality, with major toxicities including cardiac and endocrine. Overall, it was noted that cost of therapy is still high, but long-term survival is similar between transplant and standard transfusion and chelation. Morbidity risk does increase as patients age, with malignancy becoming a more common toxicity than seen previously.

#### Session 3: Hydroxycarbamide Perspectives

Moderator: Dr. Russell Ware, Cincinnati Children’s Hospital Medical Center, US.

Speakers: Dr. Russell Ware.

Ms. Yemi Moses.

Dr. Marianne Montalembert.

Dr. Patrick McGann.

In this session, Dr. Russell Ware of Cincinnati Children’s Hospital Medical Center, US moderated the discussion of hydroxycarbamide use from the US and European perspectives, with poignant testimony from a patient whose life was changed once she started hydroxycarbamide.

Hydroxycarbamide can be considered polarising regarding dose, response and laboratory toxicity. Hydroxycarbamide’s benefits included myelosuppression, elevated foetal haemoglobin, improved red blood cell (RBC) rheology, Nitric Oxide donation, reduced adhesion, and endothelial effects. Since the proof of principle and early use by Dr. Charache in the 1980’s, the timeline of hydroxycarbamide continues to proceed forward. The NHLBI consensus guidelines note that for adults, it is recommended that we educate, treat and refer to a consultant, whereas in children, it is recommended to educate, offer treatment and use a specific treatment protocol. However, there are considerable differences between how physicians in the U.S. treat patients compared to Europe, including approach, indication, goal and optimal dose, when therapy should start, and degree of monitoring needed for patients.

Ms. Yemi Moses, an SCD patient that Dr. Ware has known for many years, provided her own testimony on the benefits of hydroxycarbamide from her perspective. She noted that there were six children in her family, four of whom had SCD. She described her early childhood and the experience of having a stroke, and the therapy she needed with chronic transfusion therapy. Over time, she developed multiple allo-antibodies and could no longer receive transfusion therapy. Hydroxycarbamide was offered to her as an alternative therapy. Now a clinical research assistant, she noted that hydroxycarbamide’s effect has been studied and she has been taking it for the last 25 years. She struggled with taking it and had complications related to not taking it. She has a healthy two-year-old daughter, but she lost her sister in 2017. Ms. Moses also lost her brother to SCD. Her youngest brother with SCD underwent HSCT in 2018, and he no longer shows signs of the disease. She recommended that practitioners provide support and systems in place to care for patients.

Dr. Mariane de Montalembert described the European experience with hydroxycarbamide, which was approved for use in Europe in 2007. It was noted that although there are more patients who are on it than previous, more people should receive it than are on it. In addition, while there is consensus in the U.S. to start treatment early, in Europe and in Africa, there is no such consensus. In Europe, hydroxycarbamide is offered because of severe disease, or specific indications, including after transfusion with elevated TCD, baseline severe anaemia, renal impairment, hypoxia, silent infarct, or conditional velocity with TCD. The ESCORT trial is a long-term study of hydroxycarbamide in 1910 patients. In the study, patients were seen twice yearly with labs done every 2.5 months on average. Three-fourths of patients started hydroxycarbamide secondary to pain, while ¼ started hydroxycarbamide for off label toxicity, including renal, neural, anaemia, or other organ protection. Doses were escalated primarily in children while adults usually received a fixed dose. Reasons for modification included low counts or lack of efficacy; in children, it was primarily bone marrow toxicity, in adults it was patient choice. As related to pregnancy and hydroxycarbamide use, 115 pregnancies occurred, leading to 58 healthy children, and 28 terminations. Cryopreservation in men is becoming more used. Hydroxycarbamide safety and tolerability were good, and side effects were mild. However, long term efficacy and tolerability, as well as an optimal schedule of administration, targeting maximum tolerated dose (MTD) or optimised dose is under investigation.

Dr. Patrick McGann of Cincinnati Children’s Hospital reviewed the U.S. experience of hydroxycarbamide. Hydroxycarbamide dose escalation is based on weight, time and effort to get to MTD, and medication adherence time to discontinuation was 200 days on average, suggesting that it is not prescribed well, and patients don’t take it well. When and how it is offered and discussed is important, in that very few patients refuse hydroxycarbamide if the conversation starts at the first visit and continues throughout. Dosing can be highly variable and the time it takes to achieve MTD can take several months using a standard approach. As a result, the Therapeutic Response Evaluation and Adherence Trial (TREAT), wherein patients are given a single 20 mg/kg dose of hydroxycarbamide in clinic, with blood samples taken for pharmacokinetics at 15, 60 and 180 min to develop a population PK model, was developed. Based on this information, patients’ starting dose was almost 30 mg/kg and time to MTD was 4 months rather than 7.8 months. Sometimes, the start dose was the MTD. Based on this patient data, a foetal haemoglobin goal of 20% may be too low, and a potentially higher goal foetal haemoglobin may be necessary. In adults, the dose range is wide and foetal haemoglobin elevation is modest. Variability is multifactorial, but drug exposure is important. Hydroxycarbamide is a very good medicine, and everyone with sickle cell anaemia should be on it if not on other therapy; individualised therapy may produce a more robust response.

Additional areas of research necessary to clarify the benefits of hydroxycarbamide include pregnancy and the effects of hydroxycarbamide on mother and foetus, the use of hydroxycarbamide in non-SS genotypes, and long-term effects of hydroxycarbamide (SCCRIP). A new protocol to determine the effects of hydroxycarbamide by exposure (HOPS), a dose optimisation study that has an aggressive dosing strategy with lower toxicity thresholds and a focus on organ protection is in development. Global questions and challenges regarding hydroxycarbamide in resource rich areas include what indications and dose, adherence concerns, effects on the CNS, reproductive effects, long-term toxicity and benefits, and benefits in patients with non-SS genotypes. In low resource areas, the concerns are centred on safety, dosing, availability and access, a commitment to use, research and pharmacoeconomic solutions.

#### Session 4 – educational sessions

Speakers: Dr. Wally Smith, Virginia Commonwealth University, Richmond, Virginia.

Dr. Kalpna Gupta, University of California, Irvine.

Dr. Ify Osunkwo, Atrium Health, Charlotte, North Carolina, US.

Dr. Wally Smith, senior author and lead investigator of the Pain in Sickle Cell Epidemiology Study (PISCES) discussed pain in SCD. The old paradigm suggests that RBC sickling leads to vaso-oclusion leading to pain and utilisation, ultimately leading to mortality. However, this is not what we see in practice. Several types of pain serve as a window into the underlying pathophysiological neural mechanisms of pain, and can be a guide for developing personalised pain medicine. To help patients keep from developing chronic pain, patients should be on a disease modifying, anti-sickling agent (DMASA). Acute and chronic noncancer pain, lesions to the somatosensory nervous system leading to maladaptive plasticity within the nociceptive system were described. The recent definition of chronic sickle cell pain as defined in the literature was also reviewed. Using those definitions, sickle cell patients only utilize the health care system on about 3% of days. However, the PISCES study showed that 50% of patients have chronic pain by the definition of sickle cell pain, and about 30% of patients have pain on greater than 95% of days. Acute pain tends to be acute on chronic, mostly in the back and legs. Men and women treat their pain differently, but prayer and alcohol use were the most common coping mechanisms. Patients worried how they would be cared for as they were trying to decide whether to seek care or stay home. What is noted is that opioid use is a crutch, but patients balance analgesia with function versus dysfunction after taking them. Work productivity is down, patients become anxious and they catastrophise and somaticise. The more pain patients have the more opioids they use, but that hydroxycarbamide only seems to work with high responders. Overall, pain in SCD is generally acute on chronic, multifocal, ischaemic and inflammatory that transforms into a central/peripheral neuropathy by an unclear mechanism, leading to patients’ physical, emotional, and mental dysfunction.

Dr. Kalpna Gupta presented data on opioid use and adverse outcomes in SCD. The background behind the use of opiates in the treatment of pain and the pathophysiology of opiates on several areas of the body, including the development of hyper-analgesia and the potential risk of retinopathy, wound healing, vascular permeability, angiogenesis and haemorrhagic stroke were reviewed. Concerns regarding fear of addiction and overdose were low until the recent opioid epidemic in the United States and other areas. With over 49,000 opioid deaths in 2017 in the U.S. alone, negative attitudes developed, so that over 50% of ED physicians and 20% of haematologists think that > 20% of sickle cell patients are addicted to pain medicine, leading to a hard stop of 90 mg morphine equivalent dose (MMED) in the CDC guidelines. Hydroxycarbamide improves survival and VOC, but it does not help chronic pain. Chronic opioid therapy is the mainstay of analgesia, and even curative therapy does not eliminate pain, so the question becomes does opioid use influence survival and cause hyper-analgesia. Berkeley sickle mice and control mice randomized into two groups were treated with morphine or saline given every day. Their survival, spontaneous pain response, and evoked pain response were measured. The results showed that AA mice treated with morphine had a lower survival than saline treated AA mice; however, morphine treated SS mice had similar survival compared to saline treated SS mice. There was a significant increase in pain when morphine wasn’t given in AA mice treated with morphine. The conclusion was that chronic morphine treatment does not affect survival in SS mice; morphine can cause hyper-analgesia but does not cause tolerance to pain.

Dr. Ify Osunkwo spoke on transition from paediatrics to adult care. Transition is not a new concept in SCD, but chronic disease in the population is a recognized concern. The young adult brain has not and will not change, and the most important problems are psychosocial in nature. Paediatricians are not trained or equipped to treat “adult problems” and medical haematologists are not willing to care for child-like adults, leading to a mortality three times higher than in younger children or older adults. Adult patients are not doing well because of poor transition; health care during transition should be responsive and appropriate for what patients need. The current American Academy of Pediatrics (AAP) and NHS guidelines on transitions, include using shared perspective and sharing perspective, and that transition should continue until the patient reaches maximum potential. Barriers are access-related and psychosocial, especially considering that the adolescent brain is still developing to age 25. Our adolescent/young adult (AYA) population need to be persuaded to do what we want them to, but that there needs to be support from the community and it has to start early. The Sickle Cell Trevor Thompson Transition Project (ST3P-UP) is a program where patients were transitioned with or without a peer mentor. The initial part of the study was to determine how well each clinic followed their own model for transition, addressing key areas including a medical summary and meeting the needs of the patient, including does the patient have support, can they manage stress, do they have a pain plan, including for the ED and at home. This study validated that it is possible to change the system and teach to the process, and that while scores can improve over time, it takes time to put the project in place. It is important to teach self-efficacy and independent living skills and engage early in the process.

### Patient Advocacy Group Project

EuroBloodNet and ASCAT jointly sponsored a patient advocacy group project called “Research priority setting workshop” to address the top 10 research questions that patients wanted to be addressed. The Oxford Blood Group facilitated the workshop. There were 28 patients from 8 European and 5 Non-European nations. Patients met the day before the workshop for several hours to meet and get to know each other. On the day of the workshop, there was an individual brainstorming session, with ideas written on large post it notes. Post-it notes were grouped into themes, which were then turned into research questions agreed by the group. Each individual was given 10 small post-it notes each representing €/$/£100 million to spend on research and were asked which questions each patient would prioritize. The top 10 questions were chosen according to number of votes. The final top ten in order from most prioritized to least include:

1. How Can We Find a Universally Available Cure for SCD? (17 votes)
2. How Can We Improve QOL For People with SCD (12 votes)
3. What Are the Implications of SCD for Work or Education and How Do We Advise People, Employers and School? (10 votes)
4. How Can We Manage Acute Pain to Prevent Hospitalization? (10 votes)
5. How Do We Harness the Political Agendas to Improve SCD? (9 votes)
6. How Does Staff Diversity or Lack of Diversity Affect the Way They Face Racism, Discrimination and Stigma? (9 votes)
7. How Can We Optimize Pregnancy in SCD? (9 votes)
8. What Complementary Therapies, including cannabis and CBD oil, Work in SCD? (9 votes)
9. How Do We Manage Chronic Pain? (9 votes)
10. What Is the Impact of a Comprehensive Care Team on Outcomes with SCD? (9 votes)

### Day 2

#### Session 5 – Thalassaemia

Moderator: Dr. Ali Taher, Naef K. Basile Cancer Institute, Beirut, Lebanon.

Speakers: Dr. Ali Taher.

Dr. Maria Cappellini, University of Milan, Italy.

Dr. Antonio Piga, University of Torino, Italy.

Dr. John Porter of University College London Hospitals, UK.

This session reviewed specific topics related to beta thalassaemia.

Dr. Taher reported on the medical complications in beta thalassemia. The medical complications of transfusion dependent (TDT) or non-transfusion dependent (NTDT) patients vary based on age of the patient, degree of anaemia, and splenomegaly. TDT patients typically have more severe complications related to iron overload, and the profile of morbidity and mortality as patients advance in age has changed. Malignancy and cardiac concerns have increased, and current epidemiologic data suggests that malignancy may be 20% higher than in control groups. TDT patients may be more at risk for solid tumours, while NTDT patients may have a higher incidence of haematologic tumours. Solid tumours, including cancers of the thyroid and liver, for which environmental and behavioural causes may play a role, could be linked to iron overload. In patients with hematologic tumours secondary to immune regulatory imbalance or hydroxycarbamide effect, surveillance and screening is required yearly.

Patients with splenectomy, NTDT, and beta thal intermedia patient with high platelet count, transfusion naivety and elevated NRBC count are more likely to develop thrombosis. Splenectomised patients with a low haemoglobin also had silent infarcts. Transfusion therapy, aspririn treatment and thromboprophylaxis of any medically ill patient may play a role in preventing silent infarcts. Both TDT and NTDT patients develop renal dysfunction and proteinuria because of chronic anaemia, iron overload and tubular dysfunction, which can progress to renal failure. Patients may also develop pulmonary hypertension, and while the exact mechanism is not clear, patients with a higher nucleated RBC, higher LIC, age, history of splenectomy and nontransfusion dependent are more at risk. Treatment is primarily based on tricuspid regurgitant jet velocity; right heart catheterisation is not required to implement therapy. Novel therapies could lower transfusion burden and improve iron overload to reduce mortalities. Until new therapies are available, patients need conventional therapy for optimal disease management; older patients with comorbidities should continue to see their primary physician. Individualized therapy with appropriate transition helps patients navigate their clinical concerns and their educational, employment and social needs, including sexuality and fertility concerns.

Dr. Maria Capellinini discussed novel therapies in thalassaemia. Thalassaemia is an imbalance of the alpha/beta ratio, which is fixed with bone marrow transplant; gene therapy is a drug therapy, not a process. The published data for Zynteglo lead to approval for patients with beta thalassaemia intermedia and major who were a transplant candidate without an available HSCT match. Conditioning regime and usefulness of apheresis need to be resolved. Other gene editing techniques, now in clinical trials, reactivate foetal haemoglobin by targeting BCL11A. Other approaches target ineffective erythropoiesis through the JAK-STAT pathway or HIF2a pathway. Ruxolitinib reduced spleen size but did not increase haemoglobin, leading to its use in patients with splenomegaly preoperatively or for splenic reduction. Luspatercept, a TGF-beta ligand that promotes erythroid maturation, showed an increase in haemoglobin of at least 33% from baseline and a reduction in transfusion during any 12- and 24-week interval in phase 2 studies. There was also a reduction of iron accumulation, but there was a high risk of thrombosis. A phase three study is ongoing. Overall, several therapies are in development, with promising gene therapy and non-gene therapy approaches available.

Dr. Antonio Piga addressed the issue of fertility and hypogonadism in patients with thalassaemia. There are many data on hypogonadism, but few data on fertility. Between 1980 to 1999, among women with transfusion dependent thalassaemia, there was a 43% failure rate in women who wanted to have a child. In 2000, 35–45% of men and women with TDT wanted to have children and almost all of them were able to have children; the majority of men needed treatment. For people with NTDT, most men and women wanted children and almost all of them were able to have children naturally. Care improvement lowered severity prevalence. Iron overload leads to hypopituitarism causing low FSH and LH, leading to low levels of oestradiol and testosterone, a process that is reversible with appropriate chelation therapy. Iron may not be directly damaging to gonads, as the diagnosis of infertility is made later than hypogonadism. Studies have shown that anaemia and iron toxicity is early, as is anterior pituitary toxicity. In thalassaemia intermedia, the anaemia is early, but iron deposition and anterior pituitary damage is later, leading to later onset hypogonadism; infertility is later still. For women, because pre-pregnancy planning is a complicated process, management involves the integration of multiple specialists, including endocrinology, gynaecology, haematology and others, whereas men may need only an andrologist and haematologist. Men also may have more intact gonads. Starting and optimizing chelation and preventing early damage allows normal development; no or poor chelation results in hypogonadism and infertility.

Dr. John Porter spoke regarding chelation therapy in thalassaemia. Choice of chelation is determined by when transfusion started, where the iron is, and underlying disease. Choosing mono- or combination therapy depends on iron loading rate, ferritin, liver iron content (LIC), myocardial iron load, organ function and plasma iron levels, tolerability issues and psychologic issues. Major challenges to adequate iron chelation include adherence and dose limiting toxicity. Higher doses lead to improved iron mobilisation but must be balanced with increased toxicity. Deferiprone (DFP) and deferoxamine (DFO) remove iron from the heart better than deferasirox (DFX). Heart function improves faster than the reduction of heart iron content (HIC). Combination therapy can be administered simultaneously or sequentially. Combination therapy works better than monotherapy; either grouping of DFP + DFO or DFP + DFX can be a useful approach. DFO + DFX is a better approach to reduce LIC; the set of DFP + DFX lowers HIC more effectively. Arthralgia and gastrointestinal toxicity have been higher with DFX because of the higher doses used. Adequate iron chelation depends on adherence, tolerability, and sufficient dosing, while optimal dosing depends on what the patient is taking, tolerability, location of iron burden, and cardiac function.

#### Session 6 – curative therapies for Haemoglobinopathies (part 1)

Moderator: Dr. Julie Kanter, University of Alabama at Birmingham, US.

Speakers: Dr. Julie Kanter.

Dr. Lakshmanan Krishnamurti, Children’s Healthcare of Atlanta, US.

Dr. Julie Kanter moderated this session discussing curative therapies for haemoglobinopathies.

Dr. Krishnamurti presented “The Principle of Stem Cell Therapies in Haemoglobinopathies.” The rationale for cure is basically a risk-benefit trade-off, i.e. living without a disease while dealing with the treatment-related morbidity and mortality versus the long-term sequelae and complications of the disease. Cure is important because there is a 20-year loss of lifespan in SCD, which is the same as patients with thalassaemia. For patients with thalassaemia, the indication for transplant is transfusion dependence and a matched sibling donor. For patients with SCD, the indications for transplant are less well defined, but include history of stroke or other neurologic complication. However, 80% of transplants are done because of pain. Many patients simply want improvement of quality of life and to know what options are available to them. A newer indication is renal dysfunction.

Era of transplantation determines the success of the transplant. Before 2006 results were worse than those transplants that occurred after 2006. Other variables include the age of the patient and the type of transplant the patient had. Younger patients do better than older patients, and those with a matched sibling donor do better than those who do not. Current data for those who are young with a matched sibling donor show that patients have a 100% overall survival (OS) with a 93% event free survival (EFS). However, it should be noted that transplant results have improved in both adults and children as reported by Dr. Bernaudin. Patients with thalassaemia who are young and have a matched sibling should also undergo a BMT. Non-myeloablative or reduced intensity conditioning transplants were compared. Dr. Guilcher’s study of alemtuzumab with the standard NIH backbone of 300 cGy TBI with matched sibling donor source of stem cells showed an OS of 100% and EFS of 100% with 30–40% donor chimerism in 2 patients. Matched unrelated donor transplants have not done as well, but there has been advancement in curative therapy, especially when one compares standard therapy versus transplantation. There is now a movement toward nonmyeloablative haploidentical transplant, where the cure rate is high with low mortality rates. However, Eapen in Lancet Haematology 2019 showed that a nonmyeloablative approach was better than reduced intensity conditioning.

Gene therapy, which is a way to use genes to treat or prevent a disease by adding a healthy gene, replacing a bad gene with a good gene, or reactivating an inactive gene, such as inducing foetal haemoglobin production and thus treat both SCD and thalassaemia. The process behind CRISPR CAS 9 and the processes of BMT versus gene therapy are different. Overall, gene therapy and bone marrow transplantation need to continue to monitor long-term safety. Its benefits include relief of chronic pain, prevention of stroke, and potential prevention of ongoing organ damage. However, its cost is a deterrent, and we need to evaluate its feasibility in the rest of the world.

Dr. Julie Kanter, the moderator for this session, discussed gene therapy in SCD. The rationale for gene therapy in SCD is that mortality from this disease is too high and we need help for children and adults with this disease. The disease isn’t just about adhesion or sickling, and it is not clear how much damage is reversible. Gene therapy, as opposed to bone marrow transplant, circumvents the donor, making it attainable for any patient with SCD. Gene therapy, according to Google.com, is “the therapeutic delivery of nucleic acid into a patient’s cells as a drug to treat disease.” Gene therapy comes in two forms, gene editing and gene addition. Once the gene is inside the cell, it is added to the genome randomly. This lack of specificity is improved by CRISPR or other techniques to target where the gene should go. The cell also has two mechanisms to repair itself. The fast repair causes the gene to be mis-made, as with BCL11a, allowing foetal haemoglobin production. Homology Directed Repair (HDR) repair is where an abnormal beta globin gene is replaced by a normal Haemoglobin A gene.

In gene therapy, the lentiviral vector is the envelope that holds the letter (gene) that needs to put inside the cell. It is labelled as to where it should insert itself as well. Once stem cells are collected from the patient, they are processed with the the lentiviral vector holding its letter. Once the process is complete, the vector copy number (the number of letters that have gotten into each cell; VCN) is assessed; the transduction number (the percent of cells carrying at least one copy) is also determined. Transduction efficiency should be around 80% so that the percent of new haemoglobin is mixed into each cell, so that cells contain both types of haemoglobin for pancellular expression. In Group C of BlueBird Bio protocol Hgb-206, patients showed improved outcomes compared to groups A and B. It was noted that the VCN should not be more than 5, and it wasn’t. It was also noted that almost 100% of cells over time achieved pan cellular expression of the modified T87Q Hgb, with a normalization of haemolysis markers. Dr. Malik in Cincinnati has showed a reduced intensity regimen worked well, even with a lower VCN. There is a current study using a lentiviral vector targeting BCL11a using CRISPR technology to increase foetal haemoglobin. The risks of gene therapy include random vector insertion sites, chemotherapy and associated risks, and unintended off target effects, for which we need to look and measure. Electroporation is another way of introducing naked genes into cells. The biggest risk of gene therapy is its failure to achieve its targets. The key is to determine whether long term outcomes such as stroke and renal complications, are reversible.

#### Session 6 – curative therapies for Haemoglobinopathies (part 2)

Moderator: Moderator: Dr. Julie Kanter, University of Alabama at Birmingham, US.

Speakers: Dr. Alexis Thompson, Robert and Anne Lurie Children’s Hospital, Chicago, IL, US.

Dr. Josu De la Fuente, Imperial College Healthcare NHS trust, U.K.

Dr. Alexis Thompson, former President of the American Society of Hematology, discussed gene therapy in thalassaemia, focusing on current strategies and recent results. The current strategy of gene therapy in thalassaemia is the addition of a modified Hgb A gene (T87Q), in order to reduce transfusion independence. While other studies are enrolling, the BlueBird Bio study using Beta-AT87Q for gene therapy in thalassaemia showed that HPLC can detect the haemoglobin. Once peripheral blood stem cells were collected using plerixafor and GCSF, and after administration of chemotherapy, the product was infused. The data showed most patients achieved transfusion independence, while in those who did not achieve transfusion independence there was a 60% reduction in number and volume of transfusion compared to prior to gene therapy. There was a higher VCN at day 0, which stabilised at six months, and the modified globin could be identified. There is a fairly stable haemoglobin over 1 year out, and in long term patients, there is a stable expression pattern at least out to four years. Insertional site analysis is similar over time and there was no clonal dominance. There is a current phase 3 study in Non B0B0 patients; 10 out of 11 patients now make Hgb A T87Q. The haemoglobin is normal; there is a higher VCN number and its expression is stable. Another gene therapy study is investigating treosulfan and thiotepa as a conditioning regimen, with direct marrow infusion of stem cells so as to avoid first pass effect of the liver and spleen. In summary, gene therapy is dependent on VCN is associated with transfusion volumes; higher VCN leads to less need for blood. There are some remaining questions, including the process’s durability, modifications needed to reduce risk, its safety in younger patients, and whether the results of gene therapy trials for SCD and thalassaemia will differ or align. Ultimately, it appears that, in thalassaemia, gene therapy produces durable engraftment, TDT patients achieve transfusion independence and the safety is similar to a myeloablative approach without clonal dominance and expected side effects.

“Bone marrow therapy in paediatric patients” was presented by Dr. Josu De la Fuente from the Imperial College Healthcare NHS trust, U.K. Bone marrow therapy in paediatric patients leads to improving quality of life. Gene therapy has taken away some of the risk of HSCT. As mentioned previously, the goal is to avoid continued organ damage, and reduce harm to the liver and lungs. Standard chemotherapeutic regimens including fludarabine, treosulfan and thiotepa have become popular, keeping in mind that higher doses were not necessary as achievement of 25% donor is sufficient to cure the disease. Citing Li’s article in Blood Advances, 61% of BMT done for thalassaemia between 2000 and 2016 were matched sibling donor transplants, with age and donor making the biggest difference. There was no difference in EFS between HLA matched versus unmatched donors. As demonstrated in Dr. Cappelli’s paper in Haematologica this year, there was a higher incidence of chronic GvHD in older patients, and the risk of mortality was related to infection and GvHD. The best alternative donor for SCD patients has yet to be determined. Criteria for HSCT includes greater than 4 episodes of VOC. There is long term improvement of CNS disease with HSCT; however, posterior reversible encephalopathy syndrome (PRES) occurs in a substantially high rate. Per the EBMT experience, EFS was similar regardless of donor source (MUD, Haplo, or other). Several groups have tried to apply the Vanderbilt experience based on Johns Hopkins/NHLBI regimen through the Vanderbilt Global Transplant Consortium for Cure. Studies with this regimen have shown that in thalassaemia there are less alloreactive complications, whereas in SCD, thiotepa was the optimal arm. However, this is not the only approach; two other groups are working on lymphodepletion but have a 20% rejection rate. With SCD, there is a low risk of GvHD, but there is a 12% mortality rate. Haploidentical transplant regimens using alpha-beta depletion have also been studied; however, notable complications include macrophage activation syndrome. Ultimately, transplant is feasible and effective, but we are still learning the right cohort, determining how to stratify patients and identify them at an early age.

#### Session 7a – chronic organ damage in SCD (part 1)

Moderator: Dr. Raffaella Colombatti, University of Padua,Italy.

Speakers: Dr. Elizabeth Klings, Boston University, US.

Dr. Claire Sharpe, King’s College Hospital, London, UK.

Raffaella Colombatti moderated this important session on chronic organ damage.

Dr. Elizabeth Klings discussed pulmonary hypertension, cardiac dysfunction, and venous thromboembolism in SCD. Approximately 1/3 of patients with SS disease and 28% of patients with Hgb SC disease have an elevated TR jet velocity, and with a level of 2.5 m/s TRV, the mortality risk is 40% at 40 months, with an 83% 2 year survival. BNP and six-minute walk length also affect survival; indeed, a BNP of 160 or greater has a 78% PPV of worse outcome; a 6-min walk distance of less than 300 m is also a sign of poor prognosis. In paediatrics, when diagnosis may be difficult, MRI may be helpful. In adult patients, a TRV jet greater than 2.5 does not mean you have pulmonary hypertension; a right heart catheterisation (RHC), measuring right atrial pressure, right ventricular pressure, pulmonary artery pressure, mean pulmonary artery pressure (mPAP) and PA wedge pressure (PAWP) are necessary to diagnose pulmonary hypertension. In patients undergoing RHC, about 69% of patients did not have pulmonary hypertension, whereas 31% did, and of those, 39% had pulmonary arterial hypertension and 61% had pulmonary venous hypertension. In most patients, elevated cardiac output causes a delay of symptoms, but when they have symptoms, it is a risk factor for death. If patients had an mPAP of 25, there was a 40% 6-year mortality, with right ventricular failure; lower right heart ejection fraction was a mortality risk. Recent guidelines, however, redefined pulmonary hypertension as any mPAP greater than 20, because a normal mPAP is around 14. With progression of symptoms, a patient can be diagnosed with pulmonary hypertension based on symptomatology and follow up RHC. Per the American Thoracic Society guidelines, treatment for pulmonary hypertension include maximizing hydroxycarbamide and considering chronic transfusion therapy. The patient had developed cough all the time with hoarseness, had PND and orthopnoea, using O2 continuously. With changes in the patient’s therapy, she is now off oxygen and has an improved six-minute walk distance.

SCD is itself a hypercoagulable state, and there are endogenous sources and exogenous sources for clots. Patients with SCD can have in situ clots, large vessel thromboses, DVT, catheter related thrombosis and chronic non-occluding thrombosis, which has an incidence of 17% in severe disease, and a recurrence rate of 31–37%. There is a three times higher risk of death, and treatment with warfarin has the highest risk of treatment failure. There is a 24% incidence of DVT with a 49% recurrence rate, and a 14% rate of catheter-associated thrombosis. History of splenectomy and elevated white blood cell count are factors that increase a patient’s risk of thrombosis. Overall, in a Brazilian study of 30 autopsy patients, 76% had some degree of pulmonary arteriopathy. Treatment is primarily with pulmonary vasodilators, but there are no randomized clinical trials that showed benefit, as WALK-PHASST was stopped early without showing benefit. Medication for pulmonary hypertension do not take into account pulmonary arterial hypertension of precapillary hypertension. Pulmonary hypertension is a complex heterogeneous disease with accelerated mortality, for which chronic and acute thrombosis may play a role. Physicians cannot look at TRV alone. Patients should have a right heart catheterisation if they have a TRV over 3 m/s alone or when the TRV is lower and in combination with symptoms, including hypoxia or dyspnoea.

Dr. Claire Sharpe, spoke on renal transplantation in adults with SCD. SCD affects the kidneys because of chronic anaemia resulting from sickle haemoglobin polymerisation and haemolysis. Anaemia leads to increased cardiac output and then hyperfiltration by the glomeruli. Over time, as nephrons are lost, the remaining ones have to do more of the work, so that by the third decade of life onward, the GFR declines. Microalbuminuria leads to proteinuria and supra-normal proximal tubular function leads to hyper-phosphoremia and increased creatinine secretion. Renal dysfunction is manifested in diminished concentrating ability, impairment of distal hydrogen ion processing, and haematuria and renal cyst formation. Risk factors for developing renal dysfunction include severe phenotype, frequent admissions, renal injury, and haemolytic phenotype. Genetic modifiers of the disease include coinheritance of alpha thalassaemia, and other genetic modifiers including APOL1 and BCL11a. Other modifiers of renal disease in SCD include hypertension, diabetes, autoimmune diseases and blood borne viral infections. Management of patients with sickle nephropathy include optimising (or starting) hydroxycarbamide, blood transfusion and bone marrow transplant. More importantly, patients should have adequate hydration, careful blood pressure control, ACEI or ARB therapy, and if their renal function worsens, potentially erythropoietin, dialysis and renal transplantation. The HUSTLE study showed that patients who were on MTD of hydroxycarbamide had lower GFR than other groups and in the TWiTCH study, hyperfiltration was lower in the treatment groups.

Patients who are on dialysis are 10x more likely to die on dialysis if they have SCD, and they are much less likely to receive a kidney transplant. However, patients who have SCD and are able to get a kidney transplant live longer, but not as long as patients who don’t have SCD. Graft survival is as long as in patients who do not have SCD. Patients with SCD can receive chronic transfusion therapy to try to prevent injury to the kidney, and exchange blood transfusion is better at 1–5 years compared to those patients not on transfusion, and there is a lower rate of rejection. Patients with SCD who underwent renal transplantation have similar survival as other SCD patients, and graft survival is also similar. However, monitoring for proteinuria is important. In conclusion, hydroxycarbamide is beneficial starting in childhood, and it is important to offer renal transplantation early on and consider exchange blood transfusion. There is no good renal biomarker, including cystatin C but following the trend and the patient’s urine output is important. NSAIDs are important pain killers, and there is no reason not to use them in limited amounts and for short bursts. Patients with CKD should avoid using them.

#### Session 7a – chronic organ damage in SCD (part 2)

Moderator: Dr. Raffaella Colombatti, University of Padua,Italy.

Speakers: Mr. Markus Bankes, Guy’s and St. Thomas’ NHS Trust, London, UK.

Dr. Atul Gupta, King’s College Hospital, London, UK.

Dr. Francoise Bernaudin, Centre Hospitalier Intercommunal Creteil, Créteil, France.

Mr. Markus Bankes discussed osteonecrosis and joint disease in SCD. Osteonecrosis typically occurs at the femoral or humeral head secondary to death of osteocytes leading to pain, loss of adhesion between the bone and cartilage, decreased bone strength, and collapse of the joint. All osteonecrosis leads to joint replacement, collapsed joint or not. Once collapse happens, symptoms should not determine whether a joint should be replaced; repairing the joint decreases pain and removes joint destruction. Total hip replacements are highly effective at pain relief; it is a single procedure of choice with a durable solution. Hip replacement affects a patient’s day-to-day life, requiring shared decision-making. If the patient has moderate to severe pain, night pain, groin pain, stiffness and limp, analgesics are not working, and the daily routine is difficult, patients may opt for joint replacement. Traditional attitudes regarding age of the patient, durability of the implant, surgical risk, and acceptability of patient’s current state lead to barriers to hip replacement. Having the right clinical staff is key to safe surgery. Patients should have a comfortable anaesthesiologist, and the patient should receive a perioperative blood transfusion. Uncemented fixation with ceramic on ceramic joints assures that the new hip joint does not wear out. Core decompression on selected patients for small, uncollapsed AVN or in younger patients with mild disease is possible, but the adjuncts do not address other issues. Osteonecrosis can occur in the knees and ankles, but surgery is typically unnecessary for those joints. Identifying collapse is key and the treatment should be total hip replacement. Withholding surgery is suboptimal, especially when the patients have their whole lives to gain. Ceramics should be the material of choice for hip replacements in this group; concerns about redo hip surgery are unwarranted.

Dr. Atul Gupta, Paediatric Respiratory Consultant, spoke on Asthma and SCD: Paediatric perspectives. Asthma, wheezing, and airway hyper-responsiveness are common in the general population, with recurrent wheezing leading to asthma and restrictive lung disease. Unrecognised asthma and wheezing in SCD can lead to chronic pulmonary disease. ACS is a major risk factor for sickle lung disease especially in patients who have asthma. Symptoms of acute asthma exacerbation and acute chest syndrome are similar. Diagnosing asthma in SCD is challenging because patients with SCD have regular cough in the absence of asthma, have airway hyper-responsiveness, and SCD alone can change patients’ lung function. SCD patients have a high prevalence of atopy, leading to allergic disease including eczema and asthma. Leukotrienes are elevated in patients with SCD with and without asthma and increase with pain or ACS. Lung function testing does not predict pain or acute chest syndrome, and comorbidities such as obesity, obstructive sleep apnoea, and micronutrient malnutrition can exacerbate symptoms of SCD.

Asthma is diagnosed based on repetitive wheezing, a parental history of asthma and a history of atopy, or a history of acute chest syndrome with a normal chest x-ray. Treatment for patients with SCD and asthma is based on general recommendations; there are no randomized trials with evidence of effectiveness in asthma in SCD patients. Acute therapy includes beta agonists, like salbutamol, and the cautious use of steroids. Chronic therapy is inhaled corticosteroids, leukotriene inhibitors and inhaled long-acting beta agonist. Using a three stage medication trial to determine patient response and symptom return at therapy removal, the clinician can continue what worked. For patients with obstructive lung disease, initial therapy can be montelukast, with inhaled corticosteroids if needed. For patients with chronic cough for which no other cause is identified, treatment with montelukast, a macrolide or an anticholinergic inhaler may be used. Lung disease significantly affects mortality in SCD and asthma therapy should be assessed on a case-by-case basis. Systemic steroids should be used with caution. Larger multicentre trials are needed for best practice, and a multidisciplinary approach is needed to manage these complicated patients. Patients who had a bone marrow transplant and a history of ACS should have their lung function assessed regularly.

Dr. Francoise Bernaudin discussed long-term treatment of children with SCD with abnormal TCD velocities. Stroke in patients with SCD is 221 times more common than healthy patients; using TCDI TAMV can detect patients at risk for stroke. The STOP 1 study showed a 92% stroke risk reduction with transfusion; TCD velocities greater than 160 cm/s in the external branch of the internal carotid artery were predictive of stenosis in 10.3% of patients. After three patients had strokes, the protocol recommended transfusion at the time of elevated TCD and an MRI if imaging could not find a window. Forty-six patients switched to hydroxycarbamide if they had normalised TCD and no evidence of stenosis and had a TCD every three months while weaning off transfusion. Two patients developed stenosis, but no patient had a silent infarct or stroke. Patients on the TWiTCH trial had longer transfusion duration, were older at the time of conversion to hydroxycarbamide, and follow-up was shorter. Twenty-four patients received a stem cell transplant. There were no recorded deaths or stroke. Stenosis improved and only one patient’s TCD did not normalise.

Patients that have a TAMV greater than 200 and have a haemoglobin less than six should be started on chronic transfusion therapy or repeat TCD if less than 200. If a patient’s haemoglobin is at baseline on chronic transfusion therapy and MRI/MRA shows stenosis, the patient should continue on chronic transfusion; patients with a normal TCD and no evidence of stenosis should be converted to hydroxycarbamide and continued on hydroxycarbamide if the TCD remains low. If the TCD remains elevated with evidence of vasculopathy, transplantation could be considered. HSCT seems to fix blood flow more than blood transfusion because there are no sickled cells left. There is a strong correlation between eICA and silent infarct, chronic anaemia, and male gender. Conditional TCDs have little clinical impact; patients are not transfused and TCD is re-evaluated every three months depending on the patient’s age.

#### Session 7B – Psychology Session (part 1)

Moderator: Dr. Marsha Treadwell, University of California San Francisco Benioff Children’s Hospital Oakland, US.

Speakers: Dr. Kofi Anie, North West University Healthcare NHS Trust, London, UK.

Dr. Marsha Treadwell, University of California, San Francisco Benioff Children’s Hospital Oakland, US.

Dr. Marsha Treadwell moderated the psychology session reporting on neurocognition, improving adherence with hydroxycarbamide, psychosocial concerns and innovative psychologic interventions for people with SCD.

Dr. Kofi Anie discussed “Neuropsychological screening in SCD.” Children with SCD are at risk of overt stroke, silent cerebral infarct (SCI) and other neurologic complications leading to neurocognitive deficits, educational issues, and impaired quality of life. Neuropsychological dysfunction in patients with SCD can lead to decline in neurocognitive development, academic and educational achievement.

In patients with SCD with a pre-existing history of stroke, neuropsychological complications correlate to location and size of brain lesion. Frontal lobe strokes can lead to attention and executive function problems while left hemispheric strokes can result in language and verbal deficits. Right hemispheric lesions can result in visual or motor impairment. Strokes may lead to broad and pervasive neuropsychological impairment in the areas of general intelligence, attention and executive function, both short- and long-term memory, language, visual-motor abilities, and academic achievement, including reading, writing, mathematics and spelling. In patients with SCI, frontal lobe infarcts present with attention and executive function deficits usually more subtly; general intellectual functioning deficits are based on lesion size. SCI also affects learning, particularly reading and mathematics. Patients with abnormal transcranial Doppler (TCD) ultrasounds without a stroke can have deficits in language, especially syntax in addition to sustained attention and executive dysfunction. Based on these data, patient screening should include, in addition to TCD, detailed neurological assessment and MRI/MRA. The rationale for neuropsychology in SCD relates to the high incidence of stroke, SCI, neurological complications, and educational performance.

Comprehensive neuropsychological assessments in SCD outpatient clinics is time consuming and impractical. A two-tiered approach with initial clinic screening, and, if needed, a comprehensive follow-up assessment is more practical. A systematic strategy for cognitive and neuropsychological assessments exists in the UK. For patients who have had stroke, a comprehensive neuropsychological assessment is performed annually. For those patients who have abnormal MRI or TCD findings or have general school concerns, cognitive or neuropsychological assessments should be performed. For those patients who undergo assessment, areas of evaluation include attention/concentration, language, memory, executive functioning, processing speed, visual motor coordination, general intellectual ability, and academic performance. Students should have an educational liaison, who helps facilitate and interpret neuropsychological reports, and mediate between the schools and education department. The liaison also works with the family and educational psychologists to obtain extra tuition, help with Special Educational Needs support, and helps develop an Education, Health, and Care Plan.

Dr. Treadwell discussed “Patient reported outcomes for a cohort of adolescents and adults with SCD: findings from the SCD Implementation Consortium Registry.” Implementation science is the study of methods for improving the adaptation, implementation, and translation of research findings into routine and common practice. The route from evidence to practice includes discovering a solution (hydroxycarbamide) to a medical problem, showing that solution can work in humans, proving that the intervention is beneficial, determining that it can be given reliably, and that it can improve health care. The SCDIC is the first National Heart, Lung and Blood Institute (NHLBI) research program to use implementation science to identify and address barriers to quality care in SCD by conducting a needs assessment of barriers to care, designing intervention protocols, and developing an SCD registry. The program will be done in phases, during which a needs assessment and implementation studies will be completed. Tools including PhenX, PROMIS, and ASCQ-Me will be used to gather patient demographic data and patient reported outcomes, enhancing information regarding the validity and reliability of the measures in a geographically diverse SCD sample.

The needs assessment showed that increased pain interference and decreased self-efficacy was associated with older age, lower income, disabled status, public or no insurance, increased frequency and severity of pain episodes, higher ED and inpatient utilisation and severe pain with no healthcare. Increased pain interference was also associated with female gender. Most respondents reported that they delayed or avoided going to the ED when they thought they needed care in the last 12 months but that they had a usual care provider who generally treats “a lot of patients with SCD.” Pain severity/frequency affected satisfaction with non-acute care. Individuals who were unemployed due to disability associated with SCD had lower satisfaction.

The SCDIC started designing intervention studies upon completion of the needs assessment. Because patients forget to take their hydroxycarbamide, and providers often have a lack of knowledge about hydroxycarbamide, one intervention study explored a patient and provider focused mobile app to help patients increase their adherence and help providers increase prescribing. The aims of the study were to improve patient adherence to hydroxycarbamide by addressing memory, motivation and knowledge barriers to hydroxycarbamide use and to examine and assess provider engagement and behaviours related to the use of the app for providers. Another aim was to assess the combined effects of patient and provider interventions on hydroxycarbamide and clinical care. The final aim was to evaluate the barriers and facilitators in adopting and implementing the app interventions by conducting semi-structured qualitative interviews with multiple stakeholders (patients, providers, administrators) from each site. Because of poor education and lack of access to appropriate care in the ED, another SCDIC study attempted to improve ED care, validating that patients verified, and staff had access to, appropriate diagnosis and treatment protocols, and that patient and staff satisfaction improved. In addition, barriers to care for unaffiliated patients and possible interventions to bring them to care will be determined.

The SCDIC Registry plans to identify, enrol, characterise, and retain patients with confirmed SCD into the registry and when appropriate, enrol them in implementation studies, to develop research questions of interest and to analyse the data and publish the findings. Preliminary data from the registry show inconsistent use of hydroxycarbamide, while many patients report chronic complications, including chronic pain.

#### Session 7B – psychology session (part 2)

Moderator: Dr. Marsha Treadwell, of Univeristy of California San Francisco Benioff Children’s Hospital Oakland, US.

Speakers: Dr. Jerlym Porter, St. Jude Children’s Research Hospital, Memphis US.

Dr. Jane Hankins, St. Jude Children’s Research Hospital, Memphis, US.

Dr. Jerlym Porter presented an “Overview of hydroxycarbamide adherence.” Adherence is the extent to which patients follow agreed upon treatment recommendations as prescribed by their health care provider. The consequences of non-adherence include relapse, unnecessary changes to treatment regimens, drug resistance, decreased cost-effectiveness of medical care, inaccurate clinical trial results, mortality, and economic impact, ranging between $290–300 billion/year.

Medical treatment with hydroxycarbamide has significantly improved survival and morbidity in patients with SCD. Hydroxycarbamide non-adherence is common and related to negative health and economic outcomes. Barriers to adherence include guideline recommendations (practice barriers and physician agreement), cost (especially in low-resource settings), disease frustration, ingestion issues, and beliefs about hydroxycarbamide. Providers worry about potential carcinogenic issues and adverse side effects and doubt hydroxycarbamide effectiveness, leading to under-prescribing of hydroxycarbamide. Practice barriers include lack of staff and limited time during medical visits to counsel about hydroxycarbamide. Adherence is assessed by a variety of ways, each having advantages and disadvantages. Pill counts and patient self-reporting are done in general clinical practice. Patient adherence and pill count availability are related and can be a predictor of attendance to clinic appointments.

The Wisepill/Wisebag protocol examined the feasibility of the WISE devices, which, when activated, linked with a server and client to report that the patient had taken his or her medicine while estimating the association of hydroxycarbamide adherence as measured by the WISE devices and other adherence estimates. Adherence by WISE devices showed a lower adherence than medication possession ratio (MPR) and patient self-report, while pill counts showed much lower adherence. Participant feedback reported concerns about the device and logistics that were addressed. The Exploring Adherence Monitoring in SCD: EXAMS estimated the association between hydroxycarbamide adherence as monitored by the AdhereTech device to other adherence measures and estimated the rate of consent to the study, device use, device failure and acceptability. The study compared perceived acceptability and adherence estimates of the AdhereTech device in passive mode to reminder mode. The AdhereTech device showed that when no reminder is sent, the patient often does not take the medicine. Two key needs were determining what valid and reliable tools most accurately measure adherence and how to intervene most effectively to promote adherence.

Dr. Jane Hankins, Department of Hematology at St. Jude Children’s Research Hospital, US reported on “Mobile health applications to improve sickle cell care.” Mobile Health (mHealth) is a practice of medicine supported by mobile devices with areas of applicability including accessing clinical information, communication among health care providers, communication with patients, real-time monitoring of health, remote health care, and delivery of interventions. EHealth intervention studies in SCD, including texting and mobile phone-based apps designed to improve self-management are limited. SIMON (Scheduled Instant Messaging Over Network), a daily reminder message developed by patients, showed that text messaging alone was not sufficient to improve adherence, and adolescents and young adults wanted additional tools to help manage their SCD, including SCD education, adherence text prompts, medication education, a medication log, laboratory monitoring, and positive feedback. The Intensive Training Program (ITP), an mHealth intervention designed to increase adherence included weekly reminder messages from providers, education thru the app, and in-person education suffered from low engagement, while another intervention using directly observed therapy failed to maintain adherence post-study. It was necessary to develop a multi-component intervention app for medication adherence, using a behaviour change wheel as the framework. The app needed to be customizable, geared toward children, help with memory, and include motivation and knowledge.

A needs assessment to understand the context of hydroxycarbamide utilisation using implementation science was undertaken, evaluating patient and provider barriers seeking to develop an app to improve not only patient adherence, but also improve provider-prescribing behaviour. Using the SCDIC, the team set out to accomplish the goal of conducting interventions using implementation science to address identified barriers to care. The developmental approach was a user-centred design to create an app to increase hydroxycarbamide utilisation. Steps included a design-thinking session creating a logic model of change to increase hydroxycarbamide utilisation, a survey of patients regarding health literacy, interest in and perceived benefits of using an app to increase hydroxycarbamide use, and barriers to hydroxycarbamide, semi-structured interviews with patients to determine preferences for app features to increase hydroxycarbamide adherence, and finally focus group testing.

The InCharge Health App has six functions, Reminders, Customisation, Symptom Tracker, Motivator, Communicator, and Educator, which fall under the four domains, Personalisation, Intrinsic Motivation, Social Support, and Educator. The Integration of mHealth into SCD Care to Increase Hydroxycarbamide Utilisation (mESH) goal is to increase hydroxycarbamide utilisation through improved patient adherence and supporting provider prescribing. The approach to the study is to integrate mobile health into clinical care using a patient app (InCharge Health) and a provider app (HUToolbox), specifically designed to encourage physicians to prescribe hydroxycarbamide.

The mESH hypothesis stated that among individuals with SCD who receive hydroxycarbamide, adherence would increase by 20% at 24 weeks compared to baseline. A 20% change was clinically meaningful and expected with use of other apps in SCD while 6 months to observe lab changes from hydroxycarbamide with improved adherence was expected. The first specific aim was to improve patient adherence to hydroxycarbamide from baseline to 6 months of InCharge Health use among adolescents and adults with SCD, assessing patient engagement and behaviours, and examining the improvement in clinical and patient outcomes. The second aim was to improve provider-prescribing behaviours toward hydroxycarbamide by comparing they change in self-efficacy in prescribing hydroxycarbamide from baseline to 9 months, examining clinic characteristics and provider engagement and behaviours of using the provider app, and assessing combined effects of the patient and provider mHealth interventions on hydroxycarbamide adherence and acute healthcare utilisation. Monitoring treatment adherence is one application of the current use of mHealth; however, a multi-level approach is necessary. Implementation science allows testing mHealth for proper evaluation and adaptation that accounts for context (where patients and providers are), barriers affecting all stakeholders, and the perceptions and preferences of users (patients and providers).

#### Session 7B – psychology session (part 3)

Moderator: Dr. Marsha Treadwell, of University of California, San Francisco Benioff Children’s Hospital Oakland, US.

Speakers: Dr. Hatel Bhatt, Evelina London Children’s Hospital, London, UK.

Dr. Raselle Miller, Evelina Children’s Hospital and Guy’s and St Thomas’ NHS Trust, London, UK.

Dr. Hatel Bhatt discussed “Case reviews exploring the social, emotional and cognitive functioning of children and young people with SCD.” Children and adolescents with SCD and thalassaemia can have behavioural, cognitive, and mood related difficulties. They are vulnerable to significant stress, including anxiety, depression, poor self-concept and self-esteem, difficulties with social acceptance and peer relationships, and poor school attendance, attainment, and progress. They are also at risk for neurologic difficulties, including high risk of neurologic injury due to silent cerebral infarct and stroke, and cognitive deficit, memory, processing, motor functioning, and intellectual ability. Moreover, patients with SCD and thalassaemia have challenges with managing their treatment as well, including difficulties with managing and living with a chronic health disorder, adherence to medication and managing hospital admissions. Families, including parents and caregivers, must cope with a diagnosis at birth, increased risk of isolation in the community based on shame, embarrassment and stigma, and concerns for denial and noncompliance.

Socially, patients can miss school due to hospitalisation, which can lead to missed peer relationships, delayed school progress, delay in development, and poor educational attainment and access to education. In the area of activity and social functioning, patients have difficulty feeling normal, challenges with sports and exercise, and difficulty building a support network. From the perspective of family/systemic impact, their disease can have an impact on working life, quality of family life, support and family network, and culture and religion. Three patients with SCD who were having concerns were non-adherent or had increased inpatient admissions. Each of the patients had psychosocial stressors at home, including maternal anxiety, or previous death of a sibling, or challenges at school. All three patients had concerns regarding normalcy, stress from parents and bringing about behaviours of either avoidance of appointments or increased admissions, withdrawal, nonadherence and maladaptive coping strategies. Having an interdisciplinary team including psychology and input from other services so that patients can have the best outcomes possible is important.

Dr. Raselle Miller presented “Innovative therapeutic intervention with people living with SCD – The ‘Tree of Life’.” SCD is congenital and life threatening. Patients can have recurrent pain, both acute and chronic, leading to frequent admissions. The disease can have an impact on education, employment, independence, and relationships. Depression is two to three times more common in adults with chronic health problems. People commonly report low self-esteem and feelings of hopelessness, marginalisation and prejudice, as SCD is a misunderstood and stigmatised disease. Patients need to develop effective coping mechanisms.

People with SCD are patients with problems, who are unwell, and need looking after, who because of disease severity need inpatient admissions, psychological intervention, strict treatment regimes, and welfare support. Narrative therapy approaches centre people as the experts of their own lives. People have many skills, beliefs, values, commitments, and abilities that will assist them to reduce the influence of the problems in their lives. People can re-story their identity rather than be defined by the problems that they have faced. The Tree of Life is one way to create this story. The ‘Tree of Life’ was developed by Ncazelo Ncube and David Denborough in 2006 from work in East and Southern Africa and it originated as a critique of traditional forms of therapy. The tree is a visual metaphorical representation of an individual’s life in its present, past, and future. It emphasizes the ‘second stories’ of people’s lives and helps people think about themselves not as problems, but in ways which give people hope and encouragement, strength and more connection to what is important in their lives. The discussion aims to be empowering rather than traumatising for them.

The ‘tree of life’ approach encourages participants to adopt a holistic approach to their life situations and is divided into its various components. The Roots are what you value from where you and your family come from. The Ground is your present day-to-day life, what you do and the roles you have. The Trunk represents your qualities, skills, and abilities. The Branches are your hopes, dreams and wishes; Leaves are important people in your life; Fruit are gifts from important people; Flowers are gifts to others and Storms are challenges. The Tree of Life exercise is done in a group setting; participants attend three to four weekly sessions up to two hours each. During weeks one and two, everyone creates their own tree. In week three, the group creates a ‘forest,’ in which participants share common experiences of how their trees can stay ‘rooted’ through ‘storms’ or the difficult times that life can bring. The ‘community’ witnesses the stories and can ‘gift’ a comment. During week four, participants have further discussion about the stories and the comments reviewed. Afterward there is a celebration.

The Tree of Life is promising as a therapeutic method for individuals living with SCD. It acknowledges patients’ whole life experience, including their cultural perspective, experience of trauma and stigmatisation, socioeconomic barriers, and potential feelings of isolation. Preliminary results suggest increased self-esteem and reduced stress in all who attended. It is hoped that the program could lead to better collaboration between patients and medical staff, individuals feeling less marginalised, promotion of a feeling of identity and connectedness, and that it empowers patients to retell their stories to highlight their strengths rather than their problems. The Tree of Life is a worldwide evidence-based intervention, and is an approach suitable for all, as the program takes into account cultural background and religious beliefs when working with different patient groups. It opens a space for group discussion about the positive legacy of a person’s cultural heritage, family history, and personal stories, and the program is truly patient centred. While outcome measures need to be standardised, anyone can create a Tree of Life; staff can use it as well.

#### Session 8 – global priorities: perspectives in Haemoglobinopathies (part 1)

Moderator: Biree Andemariam, New England Sickle Cell Institute, University of Connecticut Health, Farmington, CT, US.

Speakers: Dr. Ify Osunkwo, Atrium Health, Charlotte, NC, US.

Dr. Isaac Odame, Hospital for Sick Children, Toronto, Canada.

Dr. Biree Andemariam moderated the session discussing global priorities including patient specific needs for research, clinical care, screening, and advocacy throughout the world.

Dr. Ify Osunkwo presented “SCD Patient Management Strategies and Satisfaction Levels,” a review on the results of the International Sickle Cell World Assessment Survey (SWAY). The SWAY study sought to answer whether provider-determined and patient-desired treatment goals were similar. Importantly, 21% of patients did not know their genotype. Patients’ top three goals include improved quality of life, prevent worsening of disease, and improve survival. For improvement of symptoms, while less than 23% of patients were on a long term DMASA, 63% of patients were very satisfied with their treatment. Patients expressed worry about short term and long-term effects. Moreover, 64% of patients receiving opioids worry about perception. 75% of patients need other therapy. 40% of patients have greater than 5 VOCs per year. Patients’ top four strategies for managing pain included rest, drinking water, pain medications and warm baths. Approximately 78% of patients are satisfied with their therapy, but only 66% are confident in their care. Many patients seem self-reliant, so the question is whether the therapy correlates to the best intervention or any intervention. Ultimately, 63% of patients are satisfied with their treatment, but there were some long-term and short-term side effects. If 24% of patients do not get care in the ED, it is unclear if physicians are doing the right thing. Places in Africa do not use opioids, but patients still want to feel better and have a normal life. Even though a therapy is available, patients do not use it because they are worried about side effects and long-term toxicity. Finally, about 15% of patients are using complementary therapy.

Dr. Isaac Odame discussed “Overcoming Barriers to Implementing Newborn Screening Programs for SCD in Africa.” Nigeria had 30% of the patients born with SCD, but their resources are limited. Indeed, Nigeria, India, and the Democratic Republic of Congo account for 50% of the patients with SS disease; 1 in 50 babies has SCD, causing a major health problem. Mortality for this population is high because basic facilities are lacking, there is no newborn screening, which means babies present later in life, and there is a lack of awareness. Newborn screening improves mortality significantly. Pneumococcal vaccination is included in primary immunization because it benefits the poorest countries and profoundly benefits patients with SCD, including reducing the mortality in patients less than seven years of age. Children with SCD have a 20–30-fold higher incidence of diarrhoea, malaria, and pneumonia, but because of current care, patients with SCD are more likely to survive the first five years of life. How countries handle surviving children and adolescents needing care will be critical, relying on a multipronged approach including the need for human resources, technical and educational training, advocacy in both the political arena and long-term public/private partnerships. Barriers to the program include the lack of universal national newborn screening, and the lack of public awareness and education about SCD and the value of newborn screening, stigma about the disease, and a lack of health policy makers and donor funding agencies with experience with the disease. Other barriers include the cost of testing, follow-up of screen positive babies and case management, appropriate care needs (primary care centres or sickle cell centres) and a lack of information and communication technologies. It is possible to build on existing maternal newborn screening public health service using a testing method based on cost, technical and logistical concerns that would be compact, portable and user friendly and easily stored. Point of care testing concerns include the stigma of diagnosis as a baby and lack privacy at birthing facilities. Innovative approaches are needed to integrate specialised care with primary care using a local community health nurse who is known by the community and can provide counselling while living among the community, even in the rural setting. Health policy makers need education regarding the disease and public-private partnerships need to be long term.

#### Session 8 – global priorities: perspectives in Haemoglobinopathies (part 2)

Moderator: Biree Andemariam, New England Sickle Cell Institute, University of Connecticut Health, Farmington, CT, US.

Speakers: Dr. Biree Andemariam.

Dr. Raffaella Colombatti.

Dr. Paul Telfer.

Dr. Biree Andemariam discussed the long-awaited crossroads in SCD care and whether the US is ready for a change in care. The identification of SCD in the US and the historical and social context of SCD with regard to awareness, acceptance and advocacy were reviewed. In the 1940s and 1950s, the molecular nature of SCD and its connection to foetal haemoglobin was identified. While SCD’s recognition was becoming more universal especially among the African American community by the 1960s, treatment options remained few and stigma around the disease remained high. Civil rights met this stigma head on as individuals with sickle cell disease and trait were prohibited from certain employment, and famous African Americans reported to live with sickle cell disease were silent about their condition and the disease remained without adequate advocacy. By the 1970s, enhanced advocacy across the United States lead to President Richard Nixon signing the Sickle Cell Anemia Control Act, supporting the development of the cooperative SCD centres focusing on research but also becoming centres of excellence for clinical care of Americans with SCD. Despite several therapeutic advances in the 1980s, biases and disparities persist even into present day, including potentially discriminatory practices against collegiate athletes with sickle cell trait, widespread systemic withholding of essential opioid-based pain management, and the outpacing of proper care delivery for adults with SCD by the recent palpable focus on novel SCD therapeutic development by the pharmaceutical industry.

The US struggles with abyssmal access to care as haematologists are generally disinterested in caring for adults with SCD. Disinterest is multifactorial, including poor payment and reimbursement concerns, the length of unreimbursed time for care coordination, the focus on chronic pain and the use of opioids, providers who are not ready for the socioeconomic comorbidities of their patients, and the idea that SCD is still a paediatric only disease. This has led historically to poor clinical trial recruitment, potential post-approval access problems to novel treatments, limited awareness, and a lack of patient and caregiver input. Implementation of mechanisms to sponsor and fund programming to enhance care delivery and clinical trial access and focused legislative advocacy to ensure the complete needs of SCD patients are critical.

Dr. Raffaella Colombatti from the University of Padua reported on “Rare anaemias consortium: Haemoglobinopathies.” The European Health Network and EurobloodNet have created an opportunity to allow experts across the EU to discuss rare oncologic and nononcologic tumours, and rare blood disorders, including rare anaemias including bone marrow failure syndromes. The goal of the EurobloodNet is to promote excellence, remove barriers of treatment and provide the experience of other organizations and institutions. EurobloodNet provides the tools to care for the patient including an online communication and document management site, and patients can travel to another country in the Net if the standard of care is not provided in their own country. Political and legal agreements allow members to develop a clinical patient management system so that clinicians can share patient data privately. Telemedicine services are also available. Ultimately, the clinical management system allows for virtual sharing and clinical trial management for patients with rare blood disorders. The network allows expert centers to share experiences and aims at guaranteeing equality of care for patients with rare hematological disorders in Europe, securing access to diagnosis, treatment and care for all patients, regardless of the country of residence.

Dr. Paul Telfer discussed “Thalassaemia Prevention and Treatment: Are there any models for developing countries?” Bangladesh is less developed than most other countries but has improving under five mortality. With regional differences, there is an incidence of 5–10% Hgb E, representing 3000 births per year presenting a place to learn how to manage TDT vs Hgb E beta thalassaemia. The country plans a thalassaemia treatment and screening program using a multipronged approach including nongovernmental organizations, public education and awareness, carrier screening, and prevention, prenatal diagnosis, and other measures. From a therapeutic perspective, current clinical therapies include transfusion and chelation, HSCT, and novel therapies. Other goals include research, advocacy, training and education, and genomics and personalized medicine. Although there was some assistance from UK government, the political will to establish a sickle cell and thalassaemia screening programme was done largely through public health groups and physicians. In Cyprus, patients could not get married in church unless they showed documentation of their disease status. Iran began screening couples who were planning to get married, screening men first so that women would not be stigmatised. If affected, couples could opt not to get married, and termination of pregnancy did become more acceptable. In Bangladesh, although the infrastructure is in place, testing is done only for those who have an affected child and has not yet made it to the general population. Opportunities to promote testing to the general population, including mobile phone networks, the school curriculum, and Friday morning sermons at the mosque. Women can play an important role in training of health care workers. Clinical management can be challenging, and alternative therapies, such as Luspatercept are needed. Affordability can be a large problem, leading to pharmaceutical altruism. In answer to a question, there has been an increase of births about 6–8 per year, most likely seen as a recognition of improvements of care.

### Day 3

#### Session 9 – Research Update in Haemoglobinopathies (part 1)

Moderator: Dr. Noemi Roy, Oxford University Hospital NHS Foundation Trust, UK.

Speakers: Dr. Erfan Nur, Amsterdam UMC, Netherlands.

Dr. Ambroise Wonkam, University of Cape Town, South Africa.

Moderated by Dr. Noemi Roy, this session reviewed current research in haemoglobinopathies regarding bone marrow transplantation, genomics and therapeutic interventions, next generation sequencing and the role of genetic modifiers of disease, and the development of a disease severity scoring system for thalassaemia.

Dr. Erfan Nur discussed “Adding azathioprine and hydroxycarbamide preconditioning to alemtuzumab/TBI may improve donor chimerism in matched sibling donor allogeneic stem cell transplantation in adult sickle cell patients.” Matched sibling donor transplant is the established cure for patients with SCD using an NIH-developed myeloablative conditioning regimen that yields a 95% disease free and 100% overall survival. However, the regimen has a significant chance of graft versus host disease; adults with SCD tolerate it poorly. A non-myeloablative approach including fludarabine, TBI, ATG, and cyclosporine or MMF was unsuccessful because these immunocompetent patients were not pre-treated to reduce counts or lower marrow space and oxidative stress. A nonmyeloablative approach including alemtuzumab, TBI and sirolimus resulted in four out of the 30 patients transplanted with this programme suffering from graft failure. Myeloid chimerisms were 86% donor, but only 48% donor for T cell chimerism, which persisted even after 1-year post transplant. Because recipient T cells suppression improves chimerism, azathioprine and hydroxycarbamide were added to preconditioning to reduce the T cell number and improve the marrow space. Using this regimen with unmanipulated stem cells and sirolimus for GVHD prophylaxis, ten patients aged 20–48, most with severe organ complications, were transplanted. All patients engrafted with donor phenotype. Myeloid chimerism averaged 95%, and T cell chimerisms averaged 58%, improved from previous studies. Sirolimus was tapered in over 50% of patients. Haemoglobin improved and the degree of haemolysis decreased. Adverse events included stomatitis, arthralgia, hyperlipidaemia, renal insufficiency, GvHD and autoimmune haemolytic anaemia, both treated with steroids. There was no evidence of viral reactivation or sign of macrophage activation syndrome. Hydroxycarbamide and azathioprine preconditioning prior to matched sibling donor nonmyeloablative transplant was tolerated well, revealing higher donor chimerisms in both the T cell and myeloid space. Graft versus host disease is still a risk, but improved donor T cell chimerism might reduce risk of graft rejection.

Dr. Ambroise Wonkam spoke on “Genomics of SCD and Novel Therapeutic Interventions.” SCD ontology suggests that current sequencing of all four haplotypes reveals a single origin of the S mutation in Sudan. SCD is a genetic condition with multiple complications, including lung, bone, renal and other complications. Although two medications have been approved for SCD, mortality has not changed. SCD can help as it relates to prenatal testing. Differential attitudes exist related to medical abortions for babies known to have SCD. Most parents suggest that it would be ok, but only about 40% of adult patients would consider terminating a pregnancy.

SCD can also help inform us about other genetic diseases. First, SCD is a monogenetic disease with known gene modifiers, including alpha thalassaemia; double heterozygote states lead to later diagnosis when clinical manifestations appear. Those with double heterozygotes survive longer, and pain and hospitalization rates, vaso-occlusive episodes, health care utilization and pain are related to genetic variants, including alpha thalassaemia, foetal haemoglobin, and other modifiers. Multiple genes, including alpha thalassaemia, HMOX1 and APOL1 affect kidney dysfunction; over 53 genetic variants that are relevant for kidney function are being explored. Genotype may not be the strongest predictor of hypertension, but some specific variants need to be investigated. Foetal Haemoglobin and alpha thalassaemia modify risk for stroke.

Researchers are evaluating a genomic risk model for SCD mortality using a whole exome approach. Investigators are looking at ways to affect prevention and care and evaluating Hgb F and survival. Because knockout BCL11 causes serious brain complications and erythroid enhancer problems, researchers are determining whether affecting BCL11 with CRISPR will cause any neurodevelopmental abnormalities. Eliminating BCL11 will enhance Hgb F and reduction of symptoms. What is missing is the heritability of Hgb F, such that most patients have 80–90% HBB, and Hgb F that is induced by BCL11 knockout is 10–20%. African people have 10% more genome than the reference genome, and that there is a great deal of variation and not all variants are considered.

Investing in pharmacogenomics now can help researchers identify what therapy will work in a given patient. As an example, it is known that hydroxycarbamide is only useful in 70–80% of patients. PharmacoScan is a tool that is being used to sequence genomes properly; in the US and Europe it is the same, but in Africa, it is different. The process models umbilical cord blood cells. Hydroxycarbamide works by changing microRNA. Whole micro RNA has a different expression in people on hydroxycarbamide compared to those who are not on hydroxycarbamide, with a link to foetal haemoglobin. Prenatal diagnosis is not done extensively in Africa; however, in Iran, patients will terminate pregnancy sometimes with prenatal diagnosis, reducing number of affected pregnancies. If prenatal testing is not done, numbers of new patients will be overwhelming. Information provided should be what family have requested. Most times the willingness to terminate a pregnancy is low. Families see that the care of patients in hospital is enough to let them have more children. However, 90% of families terminate a pregnancy if they have one affected patient; if they don’t have an affected child, the level will be much lower as they see a 75% chance of not having child with SCD. Dr. Wonkam was also asked about pharmacogenomics. He noted that it needs to be done properly; he and his team are still obtaining appropriate data. He noted that they may need up to 5million people throughout Africa to make sure there is correct representation of the population. When asked about inclusion of low resource countries, Low resource countries are included in Pan African conferences.

#### Session 9 – Research Update in Haemoglobinopathies (part 2)

Moderator: Dr. Noemi Roy, Oxford University Hospital NHS Foundation Trust, UK.

Speakers: Dr. Marina Kleanthous, Cyprus Institute of Neurology and Genetics.

Dr. Aurelio Maggio, Villa Sofia-Cervello. Italy.

Dr. Marina Kleanthous spoke on “NGS-based genetic diagnostic panel for inherited rare anaemias and genetic modifiers retrieved from the ITHANET portal.” A next generation sequencing based genetic diagnostic panel for rare inherited anaemias was developed to create an evidence-based gene ranking metric and a database of genetic modifiers of SCD and beta thalassaemia. An NGS panel replicated the results of candidate modifiers that affect disease severity, quality of life and response to treatment leading to phenotypic variability. For beta thalassaemia, the genotype, Hgb F level, and alpha thalassaemia coinheritance play a role. For SCD, a disease of polymerization of red cells, genotype, haemoglobin F level and alpha thalassaemia are primary modifiers. However, secondary modifiers may play a role as well. Secondary modifiers of disease phenotype other than primary modifiers were identified by association studies, evaluating family members, the patient cohort or as a case control design study. This process can be done by a candidate gene or non-candidate gene approach requiring a precise definition, a single genotype, large patient cohort, and age and sex of patients. This study requires replication and validation, followed by functional mechanistic studies once a specific causative gene is identified.

The ITHANET portal is a genetic modifier repository which contains over 300 articles online, as a database community including organizations, experts, and contributors. The database includes the Ithaverse and Ithachrome. The Ithagene database contains 297 genes and 2884 variations with 700 modifiers. The sequences include external links to the gene location, the NCBI sequence, phenotype, and publication. An ITHAScore is calculated on the number of associated functional studies and includes variations related to the gene. To date, 483 gene/phenotype integration have been scored. High or low scores show how strong or weak respectively the evidence of interaction is. For sickle cell patients suffering from proteinuria, MYH9 and APOL1 are common gene modifiers affecting renal dysfunction. All scores for genes were low or medium; existing evidence is also low. For Hgb F, there are 317 variations, with 80 SBVs for beta globin and four noncoding RNAs. For BCL11, there are 83 genes or intervening regions, and although KLF1 was evaluated no causative variation was found. Beta globin SBV was population specific, with a variation between 10 and 50%. Most gene phenotypes have low scores and replication of results is limited. To date, there are no functional or mechanistic studies. Association studies are based on candidate gene approach. Targeted NGS panel for rare anaemias and genetic modifiers is available. However, challenges include homologous regions and pseudogenes. Not all genes related to the target anaemia are known. There are large genes as well. There is no gene specific mutation database that correctly takes point mutations.

There is a need for large patient cohorts like the THALAMOS project and ARISE project. So far, 297 genes with a significant association have been identified, and the team is actively expanding the IthaGene database. Overall, the evidence of interaction is very low, and replication studies are needed. The development of a targeted NGS panel is ongoing, but large cohort studies are needed to complete the studies.

Dr. Aurelio Maggio presented “Evidence for three distinct classes of phenotype severity in beta-thalassaemia.” A severity scoring system for patients with beta thalassaemia was developed. Patients with transfusion dependent and non-transfusion dependent thalassaemia present on a spectrum. A reliable tool would be beneficial for timing and intervention to prevent complications. A collection form was implemented and information placed in a repository started in 2017 found at www.sanitacicilia.eu/IWG. The international thalassaemia repository includes several countries from the Middle East, Europe and North America.

The severity scoring system is used to institute timely intervention. The patients were evaluated and for each category was scored either a 0 for no complications or a 1 for 1 or more complications. Categories evaluated were age at diagnosis, age of first transfusion, age at first chelation, mean ferritin, AST, and ALT. Patients were divided into two categories. Group A included TDT and transfused non-transfusion dependent thalassaemia patients, and Group B included non-transfused patients. Types of complications reviewed include cancer diagnosis, cardiac damage, diabetes, hypogonadism, hypothyroidism, liver damage, and osteoporosis. The prognostic score was built by using a multivariate conditional logistical regression model for all complications. In group A, age, age at diagnosis, and haemoglobin were evaluated. The development of the scoring was completed based on the important clinical variables and the risk score divided into low, intermediate, high, and very high groups. It was noted that in the high category, there were many complications; there are less complications than in the very high group, but there is a lower number of patients in the very high group. The same approach was taken with the non-transfused patients using the same model, risk factors and raking system.

The scoring system will allow clinicians to determine patient status and classify disease subgroups. Using the scoring system can drive treatment decision making and monitor patient progress. Only beta thalassaemia patients were included. Previous splenectomy reflects severity in the past, and generally can now be delayed. It should be considered a risk factor.

#### Session 10 – plenary session

Moderator: Dr. Rachel Kesse-Adu, Guy’s and St Thomas’ NHS Foundation Trust, London, UK.

Speakers: Dr. Anders Berggren, MD, PhD, AstraZeneca.

Daniel Dos Santos, Senior Biomedical Scientist, Viapath, UK.

Dr. Emma Drasar, University College London Hospital, UK.

Dr. Rachel Kesse-Adu, Guy's and St THomas NHS Foundation Trust, London UK.

Dr. Stella Chou, The Children’s Hospital of Philadelphia, Philadelphia, US.

Dr. Rachel Kesse-Adu moderated this general session reviewing recent research, pertinent SCD case-based complications, HPLC cases and transfusion medicine in SCD.

Dr. Anders Berggren, MD, PhD reported on “Pharmacokinetics of Ticagrelor in Infants and Toddlers Aged <24 Months with SCD.” There is an unmet need for medicines in young patients with SCD. Ticagrelor is an anti-platelet agent approved for use in acute coronary syndrome, myocardial infarction, and risk of atherothrombotic event. Ticagrelor has a rapid onset, with complete inhibition of aggregation, but reversible binding. It is rapidly absorbed, and highly protein bound, metabolized by CYP3A4 to its active metabolite. It is eliminated by the biliary system, with a half-life of 7 h. Ticagrelor achieves effective inhibition, enhances adenosine levels, and decreases P selectin expression. This study, HESTIA 4 is a phase I/II study of patients less than 2 years of age weighing greater than 5 kg. Subjects received a single dose of Ticagrelor 0.2 mg/kg for patients greater than six months and 0.1 mg/kg for patients less than six months and had levels monitored at one, 2, 4 and six hours and at a follow-up visit 1 week later. Two patients less than six months of age were enrolled. All patients received the same dose as required by study. Exposure was similar in all age groups, and plasma levels were similar at 4 h in all patients. One severe adverse event of bronchiolitis occurred, but no AEs were related to study drug. The medication was well tolerated, and the dose was appropriate for the upcoming phase 3 study evaluating Ticagrelor in patients six months.

Daniel Dos Santos, Senior Biomedical Scientist from Viapath, UK reviewed haemoglobinopathy HPLC cases. Many HPLC models are available; the model used and type of test (screening or confirmation of a diagnosis) is important. Confirmatory testing includes HPLC and electrophoresis. Caution is needed for interpretation of cases, as previous transfusion can affect testing results. Identification can be difficult, especially when patients have variant haemoglobin such as Sickle C, Haemoglobin Lepore, Haemoglobin G Philadelphia or HPFH/Beta0 mutations.

Dr. Emma Drasar, Consultant Haematologist, University College London Hospital, UK reviewed several thalassaemia case scenarios. Management concerns include the use of hydroxycarbamide, splenic complications and worsening haemolytic anaemia and ITP. Pregnancy issues involve partner screening, transfusion issues, baseline haemoglobin, appropriate blood sources, and iron overload, particularly as it related to cardiac concerns, alloantibodies and foetal concerns. A patient who had a history of transfusion presented with symptoms of delayed transfusion reaction with an elevated LDH, retic count and pan-reactive antibody. She started hydroxycarbamide but stopped it because she wanted to become pregnant. She had mild iron overload, but no cardiac iron. She underwent a trial of transfusion but had a prolonged admission for delayed transfusion reaction and multiple alloantibodies. Hydroxycarbamide was restarted; but she became pregnant and erythropoietin and iron was started.

Dr. Rachel Kesse-Adu, Consultant haematologist at Guy’s and St Thomas’ NHS Foundation Trust, discussed two SCD case scenarios. A patient with sickle nephropathy was difficult to transfuse. The patient was intermittently adherent to hydroxycarbamide. The patient’s GFR got worse, and Epo was added to transfusion. Using rituximab and eculizumab in SCD is reported, but the patient continued to need blood, with worsening renal function. Patients with SCD on dialysis do not do well, and most die within 5 years of starting it. They are also prone to infection, thrombosis of dialysis access, and are much less likely to get a transplant. A patient who, while pregnant with twins, had a stroke and PE, was recommended for haploidentical bone marrow transplant. It was asked whether delayed haemolytic transfusion reactions could cause more crises, but it was noted that it does not cause crises as side effect in patients who were reacting to transfusions. It was recommended to encourage hydroxycarbamide and erythropoietin.

Dr. Stella Chou, Division of Hematology, The Children’s Hospital of Philadelphia (CHOP), US presented her team’s research on “RH genotype matching for transfusion in SCD.” Transfusion matching in SCD remains problematic despite phenotype matching strategies. It is imperative to improve methods of identifying appropriate blood for patients. Alloimmunisation occurs despite Rh matched PRBCs from minority donors; nearly 1/3 of antibodies are associated with lab evidence of delayed haemolytic transfusion reactions. Blood group genotype can predict phenotype in transfused patients based on presiding antigen. There are serological discrepancies. In some Rh variants, it is possible to distinguish between allo- versus autoantibodies. Application of blood group SNPs can lead to DNA based predictions of RBC expression. Serologic typing does not distinguish differences between European and African variant alleles. All people have the same RhD and CE alleles, but some patients will be antigen + and lack the corresponding conventional allele, particularly in the Rh groups as Rh antibodies are not explained by genotype. Patients with SCD should be transfused with Black donors because 52% of African donors are D+, C-, E- K-, whereas only 3% of Caucasians have that combination. Rh genotype testing with improved matching could result in double the number of units available with double the African American donor pool, as they are more likely to have the appropriate antigens. Barriers include cost and genotyping.

The feasibility of providing Rh genotype for sickle cell patients was explored. Whole genome sequencing using DNA array was completed and whole exome sequencing, done by targeted capture by region by DNA extraction, revealed 92–100% concordance. Genotype was predicted by expanded Red Cell Antigen profile. Blood typing was done for secondary analysis. Rh allele identification for all patients with SCD and chronic transfused patients with thalassaemia was completed. Antibody identification was done using genotyped red cells. Reagent red cells were generally serologically typed. Whole exome and whole genome sequencing for this process showed proof of principle, while improving NGS technology may decrease the cost. Investigating migration patterns of RH variants in subpopulations requires evaluating the antibody rate in those groups as well.

#### GSTT grand round: a multidimensional approach to treatment of SCD

Moderator: Dr. Baba Inusa, Evelina Children’s Hospital, Guy’s and St. Thomas’ NHS Trust, London, UK.

Speakers: Dr. Alexis Thompson, Robert and Anne Lurie Children’s Hospital, Chicago, IL, US.

Dr. Wale Atoyebi, Oxford University Hospitals, Oxford, UK.

Dr. Lewis Hsu, University of Illinois at Chicago.

Dr. Alexis Thompson, past President, American Society of Hematology, presented “American Society of Hematology (ASH): Sickle cell related updates.” The ASH Sickle Cell initiative includes Access to care, Research, Global initiatives, Policy and Strategy, and SCD coalition. ASH is working to expand education and training in SCD, develop programs on innovative care and methods, and revise and expand the current NIH expert guidelines. Guidelines for transfusion support, cardiopulmonary disease, renal disease, sickle cell trait, and pain will be available in 2019 and early 2020. The ASH research collaborative supports the clinical trials network and data hub, a registry containing aggregated data and information on the natural history of SCD from a robust large-scale contemporary repository, providing a more accurate picture of the SCD population, which is essential for successful research efforts. The selection process for the SCD CTN sites is completed; a high priority for the SCD CTN sites is enrolling their patients into the data hub. The SCD coalition is made of 77 members. There is a new CBO pilot, with a transition toolkit and distilled SCD research. An SCD awareness campaign includes a minority blood donation working group on social media. ASH supports the African SCD newborn screening and early intervention consortium, haematologists from Ghana, Liberia, Nigeria, Tanzania and Zambia, evaluating screening procedures and a treatment protocol. The consortium sets minimum requirements of participation and sets standards for infrastructure and leadership studying effectiveness of newborn screening and early therapeutic interventions.

African Research and Innovative initiative for Sickle cell Education: Improving Research Capacity for Service Improvement (ARISE). Dr. Wale Atoyebi spoke about “Laboratory Skills in Haemoglobinopathies - Training the Trainers in Nigeria.” The burden of SCD disease in Africa requires newborn screening. As part of the ARISE program, a Train the Trainer program started in September 2019. This program was built in collaboration with ASH, considering current challenges and future needs. SCD is a complex disease with complex pathophysiology and multiorgan pathology with overlap of complications across the lifespan. Care has clearly improved over the years, with average lifespan in the 65–70 range in the UK, and lower in the US. Newborn screening, early penicillin use, stroke prophylaxis and hydroxycarbamide have improved care. Newborn screening is critical to start early treatment, including diagnosis education, vaccination, antibiotics and hydroxycarbamide if available. More expensive therapy such as transfusion and transplantation can be discussed, if not offered once complications develop. The highest burden of SCD in the world is in Nigeria, Congo, and India, which represent 30% of the global diagnoses, 75% of which are sickle cell anaemia, which is 1000 babies a day. In Africa, 90% of patients die before the age of 5, yet in Europe 90% survive to adulthood. In northern Nigeria, 90% of patients with SCD will not survive, so newborn screening is targeted.

The ARISE program is designed to improve research capacity for SCD health care service improvement. Funded by the EU for 5 years, 9 EU and 5 Non-EU countries are involved. The project goals are to evaluate overall outcomes and establish comprehensive care through collaboration. Programs include diagnosis, e-health technology, and laboratory diagnostics. Countries included the UK, France, Italy, Greece, Cyprus, Nigeria, US, Lebanon, and Kenya. All professionals care for SCD patients, and participate in clinical research, drug therapy, and other research. Two people from Nigeria went to the UK and the United States to learn about newborn screening. Because pathology is global, an integrated approach to skills required them to visit 20 labs, reviewing pathology lab technology and skills including in-house controls produced for urinalysis and haemoglobin determination.

Barriers for newborn screening in Africa include a lack of public health infrastructure, awareness, and education. In Africa, six institutions were evaluated, and the laboratories were reviewed. Gap analysis revealed that accreditation, automation, basic lab amenities, quality assurance, lab space and lab practice documentation needed to be addressed. A multidisciplinary train the trainer workshop with parallel interactive sessions in Nigeria was organized, with 80 delegates attending. The workshop completed screening of patients and clinical follow-up was completed if positive. Ghana’s program included laboratory accreditation, quality assurance completed by RCPath and ASH, and CME and CPD credits were given. An advocacy needs assessment was completed. The team learned from HIV screening, where there were 16,000 babies per year, and follow-up and intervention were required. A site readiness visit was completed, and screening commenced with appropriate quality assurance. Linking newborn screening to immunisation and further health care requires government engagement to complete the work.

Dr. Lewis Hsu, University of Illinois at Chicago discussed “Application of Implementation Science in SCD in Africa.” The ARISE program developed an implementation plan linking a newborn and antenatal program for use in Lebanon and Nigeria. The program learned to scale up to the need of the population and promote the integration of research findings and evidence into health care policy practice for a complex real world, using randomised clinical trials and meta-analysis to develop consensus guidelines. To scale up and disseminate the program successfully there must be readiness, capacity, and distribution capability. Stakeholders include family, medical staff, and community. Patient needs and goals should agree with best practice. Once ready to start, a program must recognise the needs/resources, identify the goals, plan the process, evaluate the outcome and sustain the process once it is established. Based on the outcome evaluation, improving the process and sustaining the work takes the process from an ideal situation to the real world. The ARISE project is evaluating current outcomes with engagement of the Nigerian Health care system, using both teaching hospitals and general hospitals. Community health extension primary caregivers are formally training traditional birth attendants, working with them using didactics, scenarios and drama to learn how to counsel families, building capacity using an iterative approach, including public health and other stake holders in planning evaluations. Using existing infrastructure involving community stakeholders allows adaptation to local needs and aligns health ministry providers and communities to disseminate information and education broadly.

#### Session 11 – Comprehensive Care for Haemoglobinopathies (part 1)

Moderator: Dr. Andrew Campbell, Children’s National Hospital, George Washington University, Washington DC.

Speakers: Dr. Jo Howard, Guy’s and St Thomas’ NHS Foundation Trust, London, UK.

Dr. Janet Kwiatkowski, Children’s Hospital of Philadelphia, Philadelphia, US.

Moderated by Dr. Andrew Campbell, this session reviews comprehensive care for haemoglobinopathies, including adults with SCD, chelation therapy, pregnancy in SCD and finally a review of red cell morphology on peripheral blood smears.

Dr. Jo Howard discussed “How I treat the older adult with SCD.” Twenty-five per cent or more of the sickle cell population is over 40. Patients who are on hydroxycarbamide and chronic transfusion were chronically hypoxic and required nocturnal O2, several had stage III CKD, recurrent leg ulcers, worsening memory, and peripheral neuropathy. Management of these patients requires a multidisciplinary team. Each patient receives a comprehensive annual review in a multispecialty clinic with a formal intervention and transition team. Specialists include neurology, orthopaedics, renal, pain, urology, and respiratory to help manage pulmonary hypertension. The data manager for the clinic identifies patients for review and a standardised note is completed in the electronic medical record. The data manager then also completes the registry information and the PA completes the letter to the general physician. The data is then transmitted to the NHS England.

Specific information regarding renal and pulmonary complications, memory, medications, vaccination status, and pain history is elicited. Testing is based on symptoms and known complications. Admissions increase in the AYA population, but then improve as patients get older. Pain history is taken rather than looking at number of admissions based on pain. Two thirds of patients have chronic pain, with 40–80% of patients on opioids. In this population, 29% of patients have pain daily, and most pain is reported as amount of pain at home per day per month. Each patient also has a pain plan, modified for increased pain when colder. Patients who have frequent or chronic pain have home opiate use for pain but are referred to a virtual pain team. Number of transfusions is also monitored for each patient.

Patients are monitored for CRD, neurologic, ophthalmologic complications, leg ulcers, and priapism along with a basic exam. Older age is associated with increased end organ damage, especially with regard to neurologic complications. For those patients with proteinuria, a renal ultrasound is ordered and referral to a urologist with experience with SCD is made. For those patients with respiratory complications, a CT chest, PFTs, sleep study and echocardiogram are ordered. For those patients who are hypoxic, nocturnal O2 is recommended. For those patients with history of cognitive decline, MRI brain is recommended. For abnormal liver function, LFTs and liver ultrasound is ordered. For patients with AVN, imaging and an orthopaedic review is recommended. As far as other complications, 40% of patients have hypertension; obesity is another concern. Reproductive concerns and endocrine complications, particularly Vitamin D deficiency, can also occur. Psychology is also available as needed for patients.

Challenges with adult patients include multiorgan dysfunction, and progressive organ failure, along with increased non-sickle cell comorbidities. This has large impact on quality of life. Patients require a high number of appointments; haematology does improve utility of clinic, and improves access to clinical trials. Overall, disease modifying therapy including transfusions should probably be used earlier and more frequently and be offered to all those who are eligible. This includes transfusions as well. Novel therapies including Endari, gene therapy and HSCT should be considered.

Dr. Janet Kwiatkowski reviewed “Oral Combination Chelation Therapy.” For patients with SCD, chelation therapy is important to maintain a safe level of iron, to prevent or remove excess iron, to reverse organ damage and to detoxify the iron that is present. Each oral chelation agent has differences in dosing and noted side effects. Indications for combination chelation therapy include severe iron overload, inability to take an adequate dose, adherence difficulties, and very high transfusion intake. Combination chelation therapy can be used with sequential/alternating versus simultaneous dosing. However, alternating dosing provides more rapid reduction and improved organ iron removal, continuous exposure and decreased side effects, and better adherence. Conversely, with simultaneous chelation, there is a risk of over-chelation, competing binding, potentially increased side effects, and worse adherence.

The combination desferral and deferiprone taken simultaneously provides a more rapid reduction in ferritin, LIC and cardiac iron, leading to improved cardiac function and prevents endocrine complications and improved survival. The combination of Deferiprone and Deferasirox improves adherence and quality of life and enhances organ specific iron removal, even though it is harder to tell if it works because there is less urinary iron, but it appears to be at least additive. There have been case reports and open label case series of DFX + DFP versus DFO + DFP. With DFO + DFP, it was given every other day, and there were no AEs and it was tolerated well, with good control and reduced side effects. With the combination of DFP + DFX, the side effects may limit a single arm open label study. Over 1–2 years, ferritin declined, iron levels were normal, T2*MRI was normal or improved, there was reversal of cardiac iron as well.

At CHOP, 9 adult patients with a high iron burden and abnormal T2* MRI were placed on combination therapy with DFX + DFP. Six patients completed a year of therapy; two patients had a nice response, with improvement of liver and cardiac iron. Noncompliant patients got worse. The GI side effects were burdensome; some patients also developed proteinuria and ALT elevations. The refusal rate was high and GI side effects were also high. In conclusion, combination therapy with DFO + DFP versus DFP + DFX shows better improvement with oral therapy rather than sub Cu and oral therapy, but quality of life is better with both groups and adverse events are not different than monotherapy.

#### Session 11 – Comprehensive Care for Haemoglobinopathies (part 2)

Moderator: Dr. Andrew Campbell, Children’s National Hospital, George Washington University, Washington DC.

Speakers: Dr. Eugene Oteng-Ntim, Guy’s and St Thomas’ NHS Foundation Trust, London, UK.

Dr. Vishal Jayakar, Kingston Hospital NHS Foundation Trust, Surrey, UK.

Dr. Eugene Oteng-Ntim reviewed “Pregnancy in SCD.” If outcomes are improved, the focus is on homes and communities where patients are coming from. One of every 2000 children have SCD. As of now, there is a large focus on screening and diagnostic programs, including antenatal screening. However, it is noted that 36% of fathers may not be screened. This needs to be a different approach from prenatal testing. However, parents do not often terminate pregnancy of an affected child. Often non-invasive prenatal diagnosis is not available in SCD. However, in a recent study, 46 patients underwent testing and 39 of those patients were diagnosed with SCD. There were no false negatives or positives. Seven patients had inconclusive tests. Globally SCD is a major problem because 75% of patients may not reach age 5. Pregnancy in this population is a major problem because it can affect both the parent and the baby, as in the case of a mom and baby with SS disease noted on preimplantation genetic diagnosis. PGD is £10,000 and can be done for an affected or first child.

Patients who are pregnant are at high risk for VTE, preeclampsia, anaemia and infection. The data suggest the risk of these complications may be six times higher than the general population but could be higher. Most patients studied have been either SS or SC genotype. The population is living longer and having babies at younger ages. Anaemia is a higher risk in SS subtype patients, and transfusion are more needed in that population. Around 20% of patients require intensive or critical care admission, and acute chest syndrome incidence is higher in pregnant sickle cell patients as well. Placental pathology is notable for evidence of VTE and infection. Another case of a patient with SC disease was screened at 17 weeks. This patient presented with preeclampsia. It was noted that SC subtype patients should be treated the same as SS patients. There is a 2.5x higher incidence of preeclampsia in SCD patients. It is recommended that penicillin and folic acid be given for pregnant patients with SCD. Exchange transfusion is under consideration. It is noteworthy that babies of mothers with SCD are at three times higher risk of intrauterine growth retardation (IUGR) than the general population; placental pathology confirms that the SCD of the mother is part of the cause for poor placental growth. Of note, Serum Papp-A, a test to evaluate for IUGR, could be used as a marker to monitor infants of pregnant SCD patients more closely.

The Royal College of Obstetrics and Gynaecology has developed guidelines for the management of pregnancy in SCD. These include appropriate vaccinations and medical advice regarding treatment options, partner screening for chronic disease, penicillin prophylaxis as needed, and iron supplementation and avoidance of chelation. It is recommended that pregnant women with SCD start thromboprophylaxis at 28 weeks onward; NSAID use is permitted until 28 weeks, after which it is not recommended. Whether to transfuse or not is controversial, but about 24% of patients are transfused by exchange blood transfusion. However, there is some question as to whether blood transfusion in this group of patients reduces mortality. If the feasibility of exchange blood transfusion is established in this group of patients, it may be offered to mothers as a treatment option. In conclusion, pregnancy in SCD leads to adverse maternal and foetal outcomes; therefore, multidisciplinary care is essential. The effectiveness of serial prophylactic exchange blood transfusion needs to be proven beneficial before it is used widely.

Dr. Vishal Jayakar was asked to review “Red Cell Morphology.” Dr. Jayakar reviewed several cases of blood smears and marrows, allowing conference attendees to participate in identifying the cases. The importance of reviewing films with microscope is helpful. Cases of patients with blood films revealing acanthocytes, poikilocytes, ecchinocytes were reviewed; in addition, several patient cases were reviewed, while discussing salient features of peripheral blood films. Although clinicians are doing smart and nifty things with SNPs, NGS, gene therapy and other things, it is still important to go back to basics and review the blood smears.

### Plenary Oral Abstract Presentations

Moderator: Dr. Baba Inusa, Evelina Children’s Hospital, Guy’s and St. Thomas’ NHS Foundation Trust, London, UK.

This final session of the conference presents the best abstracts of the conference.

### Abstract 1

Dr. Jo Howard, Consultant haematologist, Guy’s and St Thomas’ Hospital UK - Adult SCD and Dr. Baba Inusa, Consultant in haemoglobinopathies Evelina London Children’s Hospital, interim chair of the new National Haemoglobinopathy Panel, England. Dr. Howard noted that there was a service review in 2016 of sickle cell care in NHS England. A recent review has noted that there has been a lack of development of services, geographic variation of services, a lack of staff, and poor data collection. So, to correct this, NHS England established a National Haemoglobinopathy Panel, so that patients can get access to the same quality care, personalized medicine, proper data collection for the registry, community services, and expert multidisciplinary team approach. The benefits are good for the patients, providing equity in care across the country in specialist centres, getting better service for the same money.

Local teams will treat patients with minimum standards of care and be seen by the specialist at least once per year. There will be 22 teams, 10 of which will be in London. There will be 10 centres for SCD and 4 thalassaemia centres, with a national coordinating centre that will be tasked with writing guidelines and supporting specialist and non-specialist leadership. At the local level, there will be a specialist and a local coordinating centre. The National panel will also provide quality assurance and educational opportunities and a clinical reference group. The Registry will be coordinated in a way that responsibilities and duties providing information are clear, so that the final product will be beneficial to the patient. Targets will need to be completed by January and submitted to the governing board for review and approval.

### Abstract 2

Dr. Jamie Kawadlier of University College London, UK presented the abstract, “Longitudinal patterns of daily pain in children with sickle cell anaemia: association with executive function.” Determining patterns in longitudinal daily pain is hard, as providers mainly see patients in acute pain. However, it is well known that patients have daily pain that does not bring them to the hospital. Most clinical trials use hospital admissions as either an endpoint or eligibility criteria. In addition, because most patients treat pain at home, daily pain diaries can be hard to synthesize, so the Index of Pain Experience in Sickle Cell Anaemia (IPESCA), an intuitive composite index to evaluate patients’ pain, was created. As an example, a number of neuropathic pain words such as numbness, tingling, prickling, were clustered together and showed that about 80% of patients had chronic pain but were never admitted for a pain episode. Patients could use either paper-based pain diaries, or an app-based pain diary. Patients were allowed to use the app as much as they wanted with no active reminders. It is known that daily pain commonly increases with age.

The IPESCA showed marked variability, but those patients with a high IPESCA was associated with neurocognitive dysfunction. Over the IPESCA development period, 41 patients were enrolled and 24 out of the 41 used the app. 18 used it well beyond the completion of the trial period, providing the investigators 120 days of pain tracking. The study showed that 85% of patients had pain at any level, and there was no correlation to age and IPESCA score. Six patients had no pain at all during the entire study. Patients were scored based on median, lower, and median higher pain, and results were divided based on pain level and variability of pain, so that patients could be ranked as low pain and low variability, low pain and high variability, high pain and low variability, and high pain and high variability. There was no evidence that admissions could serve as a proxy for daily pain. Cognitive results revealed that there were significant differences among the groups, such that patients with a high IPESCA and high variability and those with high IPESCA performed worse in cognitive functioning than the other groups, revealing that high pain intensity and high variability are correlated to worse pain.

### Abstract 3

Dr. Muattaz Kazzam from Guy’s and St Thomas’ NHS Foundation Trust, UK presented the abstract, “Adherence to Individualized Sickle Analgesia Plans by the Ambulance and A&E Services - A Single Centre Clinical Audit.” There are 15,000 patients with SCD in the UK, with England having the highest number nationally. Vaso-occlusive crisis is the most common reason to seek medical care, representing 90% of sickle cell patient episodes. Most patients have a personalized pain plan documented in the medical record, and care can be coordinated through MyCare, where the patient’s personalized urgent care plan can be seen and followed by all professionals including emergency services. Patients who present with pain should be assessed and given pain medication within 30 min, and if not, the delay can make the patient worse, causing deterioration of the patient’s condition. An audit in order to measure adherence to the pain plan in the ambulance and acute and emergency care setting was completed.

The team performed a pre-intervention study to measure the impact. Patients were excluded if they did not have a care plan or if they did not present with pain. They recorded the type and dose of pain medication in the ambulance versus the pain plan and what the patient received in the A&E centre compared to the plan. Time of dose was also recorded in the ambulance and the A&E setting. There were 38 episodes of pain in 28 patients. Twenty-nine of those received analgesia per the plan, three were given an overdose, and six were under-dosed. In the A&E centre, there were 45 episodes of pain; 36 were given pain according to the plan, while 9 were not given as per part of the plan. Four patients were given an overdose, while 5 patients were under dosed. In 2 out of 9 patients, a clinical rationale was given for not following the plan. One rationale was that the patient was drowsy, while the second was that they were following recommendations from haematology. Average time to dosing was 2 h and 40 min between ambulance and A&E. The minimum was 50 min, and the maximum was 3 h 44 min. It is noted that ambulances lack access to the EMR and usually end up giving fixed or set doses of morphine; however, in the A&E setting, some patients were treated outside of their care plan. Some were given higher doses; some were under dosed, with the thought that some providers might not feel comfortable giving higher doses or providers had a lack of understanding regarding the severity of pain.

NICE guidelines influence timing of medication, but a significant portion of patients still receives non-adherent dosing. Ambulances currently do not have care plans available to them, but when the CMC is in place, they will be able to see it. Dr. Kazzam plans to re-audit once the CMC is in place. As far as what providers would follow, unless the patient had a letter, providers had nothing to go by, so they would routinely give standard dosing (5 mg or 10 mg) and then determine if they had a pain plan.

### Abstract 4

Dr. Dean Smyth, NHS Ayrshire and Arran, Scotland presented the abstract, “The Changing Demographics of SCD in Scotland: Implications for Health Care Delivery.” Currently, Scotland’s population is roughly 5.4 million people, and there is a low prevalence of SCD in Scotland. However, the number of patients clinically reviewed within NHS Scotland has increased year over year, with migration as the main driver of the increase. Demographic data has been gathered and patients have been identified, and both the paediatric and adult population has been increasing year on year, with another 15 newborn patients identified so far this year. The majority of patients are in Glasgow, Edinburgh, Aberdeen and Dundee, but are as far as Firth Valley. There are 118 patients with SS genotype, representing 2/3 of the patients. Otherwise, there are 51 patients with Sickle C, 6 with S Beta thal and 1 with Sickle D Punjab. There are more and more paediatric patients, the plurality of whom were born in Scotland, although that is variable. The majority of adult patients are from Nigeria and Ghana.

NHS Scotland managed several things as numbers increased, including increasing recognition, developing a clinical network for haemoglobinopathies, provided access to a support network, and provision of specialists, investigations and multidisciplinary team meetings. Programs to improve diagnosis and access to repayment include newborn screening program for early referral to specialty care, and for professionals, programs to help with acute care complications. As for patients and families, the age of patients ranges from birth to older than 50, with the total number increasing. The key is recognizing the prevalence is increasing from migration into the country and people staying. The role of the MCN within Scotland for education and health care delivery is expanding.

## Data Availability

NA

## Abbreviations

A&E: Accident and Emergency Department
AAP: American Academy of Pediatrics
ACEI: Angiotensin Converting Enzyme Inhibitor
ACR: Albumin:Creatinine Ratio
ACS: Acute chest syndrome
ALT: Alanine Aminotransferase
ARB: Angiotensin Receptor Blocker
ARISE: African Research and Innovative Initiative for Sickle Cell Education
ASCAT: Academy for Sickle Cell and Thalassaemia Conference
ASCQ-Me: Adult Sickle Cell Quality of Life Measurement Information System
ASH: American Society of Hematology
AST: Aspartate Aminotransferase
ATG: Anti-Thymocyte Globulin
AVN: Avascular Necrosis
AYA: Adolescent/Young Adult
BMT: Bone Marrow Transplantation
CBD: Cannabidiol
CDC: Centers for Disease Control
CHOP: Children’s Hospital of Philadelphia
CKD: Chronic Kidney Disease
CMC: Coordinate My Care
CME: Continuing Medical Education
CNS: Central Nervous System
CPD: Continuing Professional Development
CRD: Chronic Renal Disease
CT: Computed Tomography
CTN: Clinical Trials Network
DFO: Desferrioxamine
DFP: Deferiprone
DFX: Deferasirox
DMASA: Disease Modifying Anti-Sickling Agent
DNA: Deoxyribonucleic Acid
EBMT: European Bone Marrow Transplant
ED: Emergency Department
EFS: Event Free Survival
eICA: External Branch of the Internal Carotid Artery
EMR: Electronic Medical Record
ESCORT: European Sickle Cell Cohort Study
EXAMS: Exploring Adherence Monitoring in Sickle Cell Disease
FSH: Follicular Stimulating Hormone
FSIQ: Full Scale Intelligence Quotient
GFR: Glomerular Filtration Rate
GI: Gastrointestinal
GSTT: Guy’s and St. Thomas’ NHS Trust
GVHD: Graft Versus Host Disease
HBB: Hemoglobin Beta
HDR: Homology Directed Repair
HESTIA: The Sickle Cell Program with Ticagrelor
HGB: Hemoglobin
HOPS: Hydroxyurea Optimization through Precision Study
HPFH: Hereditary Persistence of Foetal Haemoglobin
HPLC: High Performance Liquid Chromatography
HSCT: Hematopoietic Stem Cell Transplant
IPESCA: Index of Pain Experience in Sickle Cell Anaemia
ITP: Immune Mediated Thrombocytopenia
ITP: Intensive Training Program
IUGR: Intrauterine Growth Retardation
LDH: Lactate Dehydrogenase
LFT: Liver Function Testing
LH: Luteinising Hormone
LIC: Liver Iron Content
MA: Microalbuminuria
MESH: Integration of MHealth into SCD Care to Increase Hydroxyurea Utilization
MMED: Milligram Morphine Equivalent Dose
MMF: Mycophenolate Mofetil
MPAP: Mean Pulmonary Artery Pressure
MPR: Medication Possession Ratio
MRA: Magnetic Resonance Angiography
MRI: Magnetic Resonance Imaging
MTD: Maximum Tolerated Dose
MUD: Matched Unrelated Donor
NGS: Next Generation Sequencing
NHLBI: National Heart Lung and Blood Institute
NHS: National Health Service
NICE: National Institute for Health and Care Excellence
NIH: National Institutes of Health
NSAID: NonSteroidal Anti-Inflammatory Drug
NTDT: Non-Transfusion Dependent Thalassaemia
OS: Overall Survival
PAWP: Pulmonary Artery Wedge Pressure
PFT: Pulmonary Function Testing
PGD: Preimplantation Genetic Testing
PhenX: Phenotype and Exposures Toolkit
PISCES: Pain In Sickle Cell Epidemiology Study
PRBC: Packed Red Blood Cell
PRES: Posterior Reversible Encephalopathy Syndrome
PROMIS: Patient Reported Outcomes Measurement Information System
QOL: Quality of Life
RBC: Red Blood Cell
RCPath: Royal College of Pathology
Rh: Rhesus antigen
RHC: Right Heart Catheterisation
SCCRIP: Sickle Cell Clinical Research and Intervention Program
SCD: Sickle Cell Disease
SCDIC: Sickle Cell Disease Implementation Consortium
SIMON: Scheduled Instant Messaging Over Network
SNP: Single Nucleotide Polymorphism
SPARCO: Sickle Pan African Research Consortium
SWAY: Sickle Cell World Assessment Survey
TAMM: Time Averaged Mean of the Maximum
TBI: Total Body Irradiation
TCD: Transcranial Doppler Ultrasound
TCDI: TransCranial Doppler Imaging
TDT: Transfusion Dependent Thalassaemia
TGF-Beta: Transforming Growth Factor- beta
TREAT: Therapeutic Response and Adherence Trial
TWiTCH: Elevated TCD with Transfusions Converting to Hydroxyurea
UK: United Kingdom
US/USA: United States/United States of America
VCN: Vector Copy Number
VOC: Vaso-Occlusive Crisis
VTE: Venous ThromboEmbolism
WALK-PHASST: Treatment of Pulmonary hypertension and Sickle Cell Disease with Sildenafil Therapy

## References

[1] Nardo-Marino A, Brousse V, Rees D (2020). Emerging therapies in sickle cell disease. Br J Haematol.

[2] Ali MA, Ahmad A, Chaudry H, Aiman W, Aamir S, Anwar MY, Khan A. Efficacy and safety of recently approved drugs for sickle cell disease: a review of clinical trials. Exp Hematol. 2020;S0301-472X(20):30356-8. 10.1016/j.exphem.2020.08.008. 10.1016/j.exphem.2020.08.008 PMC7442900 32841705

[3] Inusa BPD, Colombatti R. European migration crises: The role of national hemoglobinopathy registries in improving patient access to care. Pediatr Blood Cancer. 2017;64(7). 10.1002/pbc.26515. 10.1002/pbc.26515 28371007

[4] Faro EZ, Shook L, Treadwell MJ, King AA, Whiteman LN, Ivy ED, Hulihan M, Kavanagh PL, Selk S, Oyeku S, Berns SD. A National Measurement Framework to Assess and Improve Sickle Cell Care in 4 US Regions. Public Health Rep. 2020;135(4):442–451. 10.1177/0033354920935068 PMC7383758 32639897

